# ADAR1 protects pulmonary macrophages from sepsis-induced pyroptosis and lung injury through miR-21/A20 signaling

**DOI:** 10.7150/ijbs.86424

**Published:** 2024-01-01

**Authors:** Xiaojun Zhao, Jiangang Xie, Chujun Duan, Linxiao Wang, Yi Si, Shanshou Liu, Qianmei Wang, Dan Wu, Yifan Wang, Wen Yin, Ran Zhuang, Junjie Li

**Affiliations:** 1 Department of Emergency, Xijing Hospital, Fourth Military Medical University, Xi'an, China.; 2 Department of Immunology, Fourth Military Medical University, Xi'an, China.; 3 College of Life Sciences, Northwest University, Xi'an, China.

**Keywords:** sepsis, macrophage, ADAR1, A20, pyroptosis, lung injury

## Abstract

Acute lung injury is a serious complication of sepsis with high morbidity and mortality. Pyroptosis is a proinflammatory form of programmed cell death that leads to immune dysregulation and organ dysfunction during sepsis. We previously found that adenosine deaminase acting on double-stranded RNA 1 (ADAR1) plays regulatory roles in the pathology of sepsis, but the mechanism of ADAR1 in sepsis-induced pyroptosis and lung injury remains unclear. Here, we mainly investigated the regulatory effects and underlying mechanism of ADAR1 in sepsis-induced lung injury and pyroptosis of pulmonary macrophages through RNA sequencing of clinical samples, caecal ligation and puncture (CLP)-induced septic mouse models, and in vitro cellular experiments using RAW264.7 cells with lipopolysaccharide (LPS) stimulation. The results showed that pyroptosis was activated in peripheral blood mononuclear cells (PBMCs) from patients with sepsis. In the CLP-induced septic mouse model, pyroptosis was mainly activated in pulmonary macrophages. LPS-stimulated RAW264.7 cells showed significantly increased activation of the NLRP3 inflammasome. ADAR1 was downregulated in PMBCs of patients with sepsis, and overexpression of ADAR1 alleviated CLP-induced lung injury and NLRP3 inflammasome activation. Mechanistically, the regulatory effects of ADAR1 on macrophage pyroptosis were mediated by the miR-21/A20/NLRP3 signalling cascade. ADAR1 attenuated sepsis-induced lung injury and hindered the activation of pyroptosis in pulmonary macrophages in sepsis through the miR-21/A20/NLRP3 axis. Our study highlights the role of ADAR1 in protecting pulmonary macrophages against pyroptosis and suggests targeting ADAR1/miR-21 signalling as a therapeutic opportunity in sepsis-related lung injury.

## Introduction

Sepsis is acknowledged as one of the main causes of death in the emergency room, with high incidence and mortality rates globally over many years 1. Sepsis is a multifaceted disease with evolving definitions. The latest study states that sepsis is a dysregulated host response to infection that can result in life-threatening organ failure 2. Sepsis is characterized by increased inflammation as well as immune suppression, and the imbalance between them leads to cellular dysfunction and even multiple organ failure. Clinically, six organ systems, the cardiovascular, respiratory, renal, neurological, haematological, and hepatic systems, are frequently examined in patients with sepsis 3. Among them, sepsis-induced pulmonary dysfunction, which results in tachypnoea and acute respiratory distress syndrome (ARDS), is intimately associated with hypoxia and metabolic acidosis 2. ARDS is a serious complication of sepsis with high morbidity and mortality 4. Although the application of lung-protective ventilation has improved the prognosis of ARDS, additional research examining the mechanism and pathogenesis of sepsis-related acute lung injury (ALI) is also important to develop more efficient and novel approaches for ALI treatment 5.

Numerous cell death processes, such as apoptosis, necrosis, autophagy, and pyroptosis, are always activated as sepsis progresses 3. Pyroptosis is an inflammatory programmed cell death that is mediated by caspases-1/4/5/11 6, 7. The Nod-like receptor (NLR) family pyrin domain containing 3 (NLRP3) inflammasome mediates caspase-1 activation and the secretion of the proinflammatory cytokines interleukin (IL)-1β and IL-18 8-10. Inflammatory caspases cleave the gasdermin D (GSDMD) substrate, which is important for pore formation on cell membranes, cytokine release, and ultimately pyroptosis 11. Bacterial infection is a crucial incentive for the assembly and activation of the inflammasome in the host response 12. Pyroptosis is needed for defence against bacterial infection to minimize tissue damage during sepsis. However, overactivated pyroptosis can result in septic shock, multiple organ dysfunction syndrome, or an increased risk of secondary infection 8. Studies have revealed that pannexin-1 and P2X7 signalling triggers pyroptosis of bone marrow-derived macrophages through LPS-induced activation of caspase-11, suggesting possible therapeutic options for patients with bacterial sepsis 13. Additionally, Xue et al. found that miR-21 mediated nuclear factor kappa B (NF-κB) signalling and protein A20-mediated NLRP3 inflammasome-regulated caspase-1 activation, both of which were positive regulators of LPS-induced sepsis and pyroptosis 14. A prospective cohort study revealed increased peripheral blood mononuclear cell (PBMC) pyroptosis in patients with sepsis, which was related to disease severity and patient mortality; however, the mechanisms of pyroptosis in sepsis and its related organ disorders are complex, so in-depth exploration of the regulatory molecules and mechanisms of septic pyroptosis is important to develop targeted interventions and improve clinical outcomes 15, 16.

The regulation of cell death is greatly influenced by RNA modification 17. The RNA editing enzyme known as adenosine deaminase acting on double-stranded RNA 1 (ADAR1) is widely known for converting adenosine residues into inosine (A-to-I) in double-stranded RNAs 18. Studies have revealed that ADAR1 regulates innate immunity and cell death mechanisms such as pyroptosis, apoptosis, necrosis, and PANoptosis 19, 20. These processes are crucial for host survival, development, and immunity against infections. Additionally, ADAR1 can modify the precursors of miRNA as well as the mature miRNA sequence during the production of microRNAs, which suppresses the expression of downstream target genes 21. Our previous study demonstrated that ADAR1 regulated macrophage polarization by binding to miR-21 precursors and editing miR-21 biogenesis 22. ADAR1 was also found to alleviate sepsis-induced inflammation and intestinal injury in mice by interfering with miR-30a synthesis and altering the miR-30a/SOCS3 axis 23. Collectively, ADAR1 regulates the development of sepsis, although it is still unknown whether ADAR1 has an impact on the organ failure and cell death pathways induced by sepsis. Through clinical samples, animal models, and in vitro experiments, we investigated the regulatory effects and underlying mechanism of ADAR1 on sepsis-induced lung injury and pulmonary macrophage pyroptosis, offering new insights and fundamental evidence for the therapeutic intervention of sepsis-related lung injury and diseases.

## Methods

### Ethic statement

All procedures during the study were approved by the Ethical Committee of Xijing Hospital of the Fourth Military Medical University (approved number: KY20212172-C-1). The human study complied with the principles of the Helsinki Declaration, and all participants signed informed consent forms. The experiments relating to mouse models were performed according to the Guide for the Care and Use of Laboratory Animals and approved by the Institutional Animal Care and Use Committee of the Fourth Military Medical University (approved number: 20190305).

### Patients and PBMC sample collection

We mainly enrolled patients with sepsis who presented to the emergency department and intensive care unit in Xijing Hospital from 07.2021 to 07.2022. Patients with sepsis were diagnosed with a SOFA score ≥2, combined with at least one infection site, and were managed following the newly updated international guidelines 24, 25. Patients who were <18 or >80 years old or pregnant, had other blood diseases, cancer, or autoimmune diseases, and had incomplete clinical data were excluded. There were 52 patients with sepsis and 36 healthy volunteers included in the study. We collected blood samples from these patients within 24 h of admission. For PBMC isolation, blood samples were added to a Lymphocyte Separation Tube for Human Peripheral Blood (DAKEWE BIOTECH, Shenzhen, China) and centrifuged at 400 g and room temperature for 20 minutes. Plasma (supernatant) samples were collected and stored at -80 °C for further use. PBMC layers were isolated and centrifuged at 400 g and 4 °C for 10 minutes. The precipitate was added to red blood cell lysis buffer (Yeasen, Shanghai, China) and centrifuged again, followed by washing with PBS and further centrifugation three times. The final precipitate was kept as PBMC samples in liquid nitrogen.

### RNA sequencing analysis

RNA sequencing analysis of PBMCs in the study was conducted by Gene Denovo Biotechnology Co. (Guangzhou, China). Four PBMC samples from patients with sepsis and 4 from healthy subjects were randomly selected to perform mRNA transcriptome sequencing. Briefly, RNA quality was detected by an Agilent 2100 Bioanalyzer (Agilent Technologies, Palo Alto, CA, USA) and RNase-free agarose gel electrophoresis after total RNA extraction. Oligo (dT) beads were used to enrich mRNAs, which were fragmented into short fragments and reverse-transcribed into cDNAs. Double-strand cDNAs were purified using a QiaQuick PCR extraction kit (Qiagen, Venlo, Netherland), end repaired, poly (A) added, and ligated to the Illumina sequencing adapter. The products were sequenced using an Illumina NovaSeq 6000. Differentially expressed genes (DEGs) were screened by the parameters of fold change ≥ 2 and *p* value ≤ 0.05. The Kyoto Encyclopedia of Genes and Genomes (KEGG) database was used to perform pathway enrichment analysis of the screened DEGs.

### Bioinformatics analysis

Expression data of *ADARs* in different cell types were obtained from The Human Protein Atlas website (https://www.proteinatlas.org/) 26, 27. Single-cell RNA sequencing data (GSE207651) were obtained from the GEO database (https://www.ncbi.nlm.nih.gov/geo/) and analysed using the Seurat R package (version 3.0.1). Cell types were clustered and annotated as previously described 28.

### Luminex assay

Serum levels of cytokines (IL-1β, IL-6, TNF-α, IL-10, and IL-4) in 29 patients with sepsis and 6 healthy volunteers were detected by a Luminex assay using a Human XL Cytokine Luminex Performance Panel Premixed Kit (#FCSTM18-30, R&D Systems, Minneapolis, MN, USA) according to the manufacturer's instructions. Outlier data were removed during statistical analysis.

### Scanning electron microscopy (SEM)

Cell samples were rinsed with PBS, followed by fixation with electron microscope fixation liquid (#CR2202015, Servicebio, Wuhan, China) for 30 min in the dark. The slides were preserved at 4 °C. After dehydration and drying, the samples were sputtered with gold for 30 s by a sputter coater and further detected under a scanning electron microscope (Hitachi SU8100, Tokyo, Japan).

### Animal model establishment

Male C57BL/6 mice (weight: 20-25 g, age: 8-10 weeks) were purchased from the Animal Center of the Fourth Military Medical University. Before modelling, all mice were adaptively cultured under normal conditions for one week. An animal sepsis model was established through caecal ligation and puncture (CLP) as previously described 29. Briefly, mice were anaesthetized by inhalation of isoflurane. A midline incision (1 cm) on the lower abdomen was made, and then, the free end of the caecum was gently removed from the abdominal cavity to carry out caecal ligation by a 3/0 silk suture at the middle and two punctures with a 21-gauge needle, followed by gently squeezing a small amount of faeces to keep the puncture sites open. The caecum was carefully returned to the original position, and the abdomen was closed. A similar operation without CLP was performed on the mice in the sham group. A subcutaneous injection of normal saline (1 ml) was given for fluid resuscitation after surgery. A total of 1×10^8^ PFU of ADAR1-overexpressing adenovirus (dissolved in 200 μl of PBS) was administered to septic mice through tail vein injection. Lung tissue samples were collected at 0 (sham), 6, 24, 48, and 72 h after surgery. Mouse ADAR1 overexpression adenovirus (Ad-O/E-ADAR1, NM_001146296.1) was cloned and inserted into the pENT/CMV vector and prepared through a commercial service from GenePharma Co. Ltd. (Shanghai, China). Subsequent animal experiments were performed at 24 h after CLP administration.

### Complete blood count

One hundred microlitres of peripheral blood was taken from the mice and transferred into a disposable capillary tube (#KJ002, KangJianMedical Apparatus, China). The tube was gently tapped to ensure optimal contact between the blood and anticoagulant coating on the inner wall of the tube. The tube was allowed to stand at room temperature for 5 minutes and then gently tapped again prior to utilizing the fully automated blood cell DF-3000Vet Analyzer (Beijing, China).

### Determination of bacterial load in blood and lungs

Blood samples from the orbital cavity and lung tissues of mice in three groups, namely, sham, CLP, and CLP+OE-A, were obtained. Blood (100 μl) was collected from the orbital cavity of each group of mice and transferred into a sterile anticoagulant tube (#G4811-1.5ML, Servicebio). The bacterial count was determined by plating 100 μl of undiluted blood onto plate count agar medium (#HBPM002, Hopebio, China), followed by incubation at 37 °C. Subsequently, the colonies were counted. Furthermore, the lungs were excised and maintained under sterile conditions. A small volume (100 μl) of tissue homogenate was diluted 10-fold in sterilized phosphate-buffered saline (PBS), inoculated onto plate count agar medium, and incubated overnight at 37 °C. Afterwards, the bacterial colony count was determined 30, 31.

### Haematoxylin and eosin (HE) staining and scoring

Lung tissues were embedded in paraffin and cut into 5 μm-thick slices, followed by staining with haematoxylin and eosin dyes (Servicebio). The slices were observed under an upright microscope (Nikon Eclipse E100, Tokyo, Japan). The disease score and lung injury score of tissues were blindly evaluated by an experienced pathologist based on six random fields of vision of each HE-stained slice according to previously published scoring criteria with a slight modification. The disease clinical scoring system contained 5 aspects: appearance (4 points), behaviour changes at resting state (3 points) and after stimulation (3 points), respiratory clinical signs (3 points), and dehydration status (5 points) 32. Lung injury scoring mainly included 4 aspects: pulmonary interstitial oedema, alveolar oedema, inflammatory infiltration, and alveolar haemorrhage, with each criterion scored according to severity (4 points in total for each, higher points indicated more severe damage) 33. The lung injury scores were calculated by the sum of these criteria.

### Cell culture and transfection

The murine RAW264.7 macrophage line was purchased from the Cell Culture Center, Chinese Academy of Medical Sciences (Beijing, China). The cells were cultured in high-glucose DMEM (Servicebio) with 10% foetal bovine serum (Zeta Life, Menlo Park, CA, USA) and 1% penicillin‒streptomycin (Biosharp, Anhui, China) at 37℃ under 5% CO_2_. RAW264.7 cells (1×10^6^) were treated with 1 μg/ml lipopolysaccharide (LPS, Corning, Wilmington, NC, USA) to mimic the septic environment in vitro (6, 12, and 24 h). For cell transfection, RAW264.7 cells were seeded on 60 mm-cell dishes and cultured with serum-free Opti-MEM (Gibco, Brooklyn, NY, USA) 1 h before transfection when cell confluence reached 70-80%. Then, the cells were transfected with ADAR1-overexpression adenovirus (GenePharma), ADRA1-siRNA (RiboBio, Guangzhou, China), A20-siRNA (RiboBio), miR-21 mimic, miR-21 inhibitor (RiboBio) and their negative controls through Zeta transfection reagents (Zeta Life). Further LPS treatment was performed 24 h after transfection. The cells were collected for further experiments 48 h after transfection.

### Immunofluorescence (IF) staining

Immunofluorescence staining was performed on both lung tissue and cellular slices through routine operation. For tissue staining, after dewaxing, rehydrating, antigen repairing, and blocking, paraffin-embedded tissue slices were incubated with diluted primary antibodies of anti-F4/80 (#GB11027, Servicebio), anti-Cytokeratin 7 (CK7, #GB11225, Servicebio), and/or anti-ADAR1 (#SC-73408, Santa Cruz), anti-cleaved-GSDMD (#AF4013, Affinity, Jiangsu, China), TUNEL (#GDP1043, Servicebio), anti-iNOS (#GB11119, Servicebio), or anti-CD163 (#GB11340-1, Servicebio) at 4 °C overnight, followed by incubation with their corresponding secondary antibodies at room temperature for 1 h in the dark. Between double staining procedures, 488-TSA (Servicebio) incubation and reantigen repair were conducted. DAPI (#G1012, Servicebio) was used for nuclear counterstaining. The slices were mounted with antifluorescence quenching sealing reagents (Boster, Wuhan, China) and captured under a fluorescence microscope (Life Technologies, Carlsbad, USA). For cellular staining, the primary antibodies used were anti-cleaved GSDMD (#AF4013, Affinity) and anti-caspase-1 (#GB11383, Servicebio). Mean fluorescence intensity (MFI) in a random field of view was measured using ImageJ software.

### Preparation and induction of bone marrow-derived macrophages

Bone marrow-derived macrophages (BMDMs) were prepared following previously established protocols 34. BMDMs were stimulated with LPS (1 mg/mL) to induce M1 polarization, and after 24 hours, the cells were harvested for further experimentation.

### Flow cytometry analysis

Flow cytometry was adopted to assess molecular expression alterations in specific cells of human PBMCs and RAW264.7 cells in different groups. Human PE-conjugated CD14 antibody (#12-0149-42, eBioscience) was used to label monocytes of PBMCs for 30 min on ice in the dark. After three washes with FACS buffer, the cells were treated with BD Cytofix/Cytoperm Fixation and Permeabilization Solution (BD Biosciences) for 30 min and 1× BD Perm/Was buffer for 15 min. Anti-cleaved-GSDMD (#ab227821, human, Abcam, Shanghai, China) was further incubated with the cells for 30 min, followed by the addition of Alexa Fluor™ 488-conjugated goat anti-rabbit IgG (#A11008, Thermo Fisher Scientific, Waltham, MA, USA). For murine RAW264.7 cell detection, APC-conjugated anti-F4/80 (#MF48005, eBioscience, San Diego, CA, USA) was used to label macrophages. FITC-conjugated anti-CD11c (#11-0114-81, eBioscience) and PE-conjugated anti-CD206 (#12-2061-80, eBioscience) were used to label M1 and M2 polarization. Cell pyroptosis was evaluated by anti-cleaved GSDMD (#ab255603, mice, Abcam) and the following flow cytometry secondary antibody: Alexa Fluor™ 488-conjugated goat anti-rabbit IgG (#A11008, Thermo Fisher Scientific). The cell death states of BMDMs and RAW264.7 cells were determined using the FITC Annexin V Apoptosis Detection Kit I (#556547, BD Pharmingen, San Diego, CA) according to the manufacturer's instructions. In brief, cells (1×10^6^) were resuspended in 100 μl of 1× binding buffer, followed by the addition of 5 μl of Annexin V-FITC and 10 μl of PI. Subsequently, the samples were incubated for 30 minutes at a temperature of 4 °C in darkness, and the fluorescence signals were detected using a COULTER EPICS XL flow cytometer. In addition, for determination of the CD4^+^ and CD8^+^ T-cell subsets within the mononuclear cell suspensions prepared from the blood of mice, the levels of CD4^+^, CD8^+^, and CD4^+^/CD8^+^ were measured using a flow cytometer. Mouse APC-conjugated anti-CD19 (#17-0193-82, eBioscience) and PE-Cyanine7-conjugated anti-CD3 (#25-0032-82, eBioscience) were used to label B and T lymphocytes. T-cell subsets were analysed by FITC-conjugated anti-CD4 (#11-0042-83, eBioscience) and PE-conjugated anti-CD8 (#12-0081-82, eBioscience) 35. Cell samples were analysed using a NovoCyte flow cytometer and NovoExpress software (ACEA Biosciences, San Diego, CA, USA).

### RNP immunoprecipitation (RIP)

The association between ADAR1 and miR-21 was studied in our previous study. Here, we performed RIP experiments again to verify the binding effects of ADAR1 on pre-miR-21 using RAW264.7 cells as previously described 22, 36. A total of 20 million RAW264.7 cells were collected and lysed and precipitated with 30 mg of anti-ADAR1 (#SC-73408, Santa Cruz) or anti-IgG antibodies (#2729, CST) at room temperature for 4 h. IP products were then reverse transcribed, and quantitative PCR analysis was performed to assess the expression of pre-miR-21 and the internal reference.

### Dual luciferase reporter assay

A pmirGLO Dual-Luciferase miRNA Target Expression Vector (GenePharma) containing wild-type (WT) or mutant (MUT) 3'UTR of A20 (sequences are shown in the figure) was constructed and cotransfected with miR-21-5p mimic or mimic-NC into RAW264.7 cells using Lipofectamine 2000 reagent (Invitrogen, Carlsbad, CA, USA). Twenty-four hours after transfection, the relative luciferase activity of the cells was determined by an Infinite M1000 multimode microplate reader (Tecan, Männedorf, Switzerland) via a dual-luciferase reporter assay system (Promega, Madison, WI, USA).

### Quantitative reverse transcription (qRT)-PCR

Total RNA from human PBMCs, murine lung tissues, or RAW264.7 cells was extracted using TRIzol reagent (Invitrogen) and then reverse-transcribed into cDNA by a PrimeScript RT Reagent Kit (TaKaRa, Beijing, China) according to the manufacturer's instructions. Moreover, the miRNA qRT‒PCR Stater Kit (#C107R-1, RiboBio) was used for RT and qPCR for miRNA. Further quantitative PCR was performed using cDNA templates, primers, PCR Mix (#11141ES60, Yeasen), and SYBR Green Mix (#11201ES08, Yeasen). Specific primer pairs were synthesized by Tsingke Biotechnology (Beijing, China). The primer sequences are shown in Supplementary Table 1. The primer pairs of miR-21 and premiR-21 were synthesized by RiboBio. GAPDH and U6 RNAs were used as internal references for coding genes and miRNAs, respectively. Final relative mRNA expression was calculated using the 2^-△△CT^ method.

### Western blotting

Total protein samples were extracted using strong RIPA lysis buffer (GenStar, Beijing, China) with protease inhibitor cocktail (MedChemExpress, Shanghai, China). A BCA protein assay kit (Sxzhhc, Xi'an, China) was adopted for protein quantification. Equal amounts of protein samples were loaded onto gels to conduct SDS‒PAGE, which was transferred onto PVDF membranes (Merck Millipore, Darmstadt, Germany). After blocking, the membranes were incubated with diluted primary antibodies at 4 °C overnight and then incubated with the corresponding secondary antibodies at room temperature for 1 h. ECL A/B reagents (DiyiBio, Shanghai, China) were added to the membranes for chemiluminescence detection, which was observed and captured using a ChemiDoc imaging system (Bio-Rad, Hercules, CA, China). The primary antibodies used in the study were as follows: anti-NLRP3 (#15101, CST), anti-caspase-1 (#GB11383, Servicebio), anti-GSDMD (#AF4013, Affinity), anti-cleaved-GSDMD (#ab227821, Abcam), anti-cleaved-GSDMD (#ab255603, Abcam), anti-IL-1β (#12242, CST), anti-ADAR1 (#sc-73408, Santa Cruz), anti-A20 (#ab92324, Abcam), and anti-β-actin (DiyiBio). β-Actin was used as the internal reference.

### Statistical analysis

All data are presented as the mean ± standard deviation (SD). All experiments contained at least three replicates, and the exact number of sample sizes in each experiment is indicated in the figure legends. GraphPad Prism 9.0 (GraphPad, Inc., La Jolla, CA, USA) was applied for data analysis. Student's t test was used to analyse the differences between two groups. Ordinary one-way ANOVA combined with Tukey's multiple comparisons test was used to compare differences among three or more groups. The nonparametric Kruskal‒Wallis test with Dunn's multiple comparisons test was used to analyse the differences in the scoring system between groups. A *p* value less than 0.05 was considered statistically significant.

## Results

### Pyroptosis is activated in PBMCs from patients with sepsis

**Figure 1:**
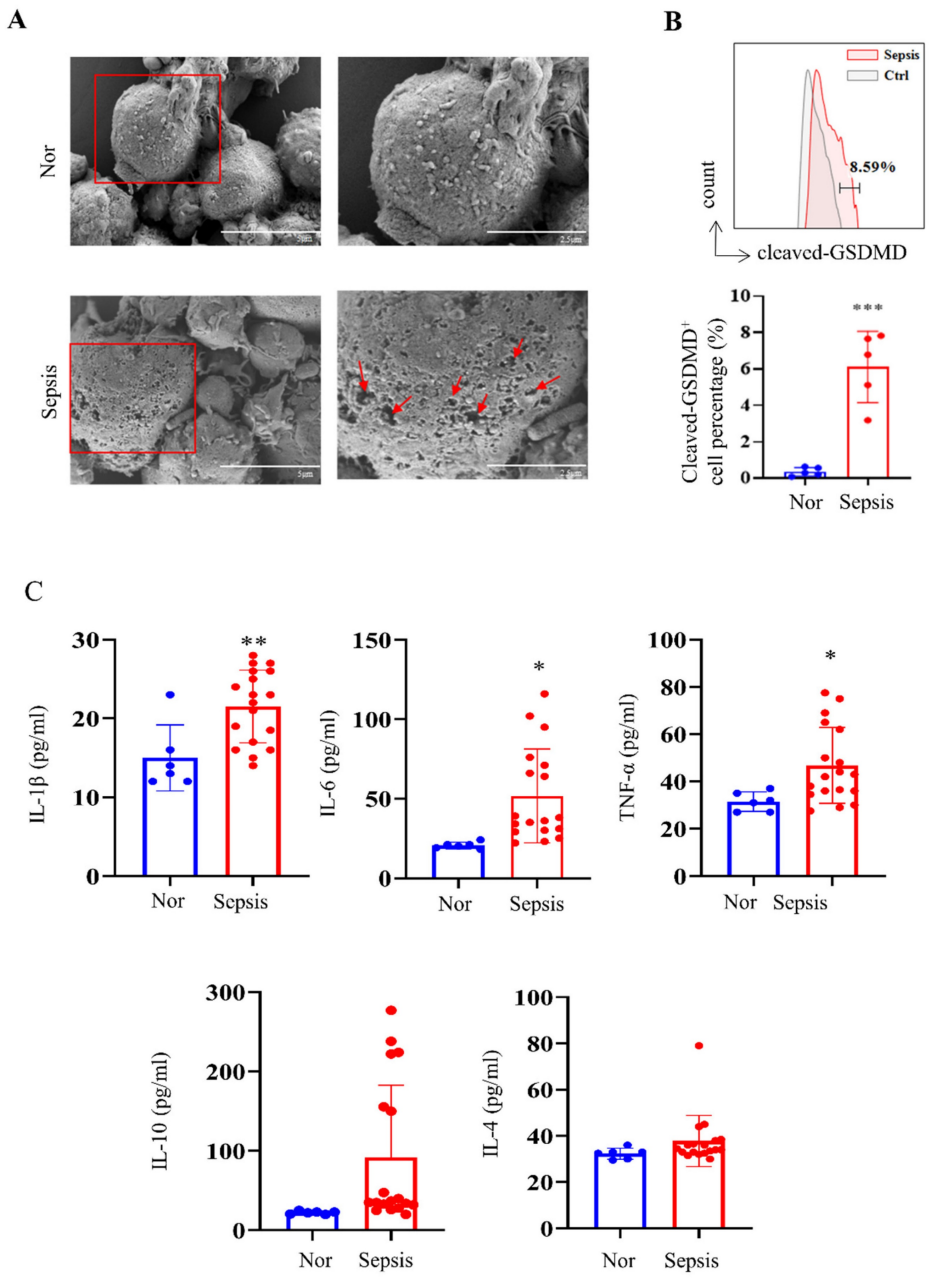
PBMCs from patients with sepsis showed a pyroptosis-like phenotype. (A) Representative SEM images of PBMCs from healthy volunteers and patients with sepsis. Scale bar indicates 5 μm and 2.5 μm. The red arrow indicates pore formation. N=3. (B) Flow cytometry showing the percentage of cleaved GSDMD-positive cells in PBMCs from healthy subjects and patients with sepsis. N=5. (C) Luminex assay showing the serum concentration of several cytokines in healthy subjects (N=6) and patients with sepsis (N=18). Data are shown as the mean ± SD. *P< 0.05, **P< 0.01 versus Nor. Nor: healthy volunteer.

We first collected human PBMC samples from healthy volunteers and patients with sepsis admitted to our hospital. Morphological analysis showed obvious cell swelling and pore formation on the cell membranes of PBMCs in the sepsis groups (Fig. 1A), which are characteristics of pyroptosis 37. We further discovered that PBMCs from patients with sepsis showed considerably higher levels of cleaved GSDMD-positive cells than those from the healthy group (Fig. 1B), which is consistent with the fact that the cleavage of GSDMD enhances the creation of membrane pores during pyroptosis. The Luminex assay revealed that while IL-10 and IL-4 levels did not show significant changes, blood levels of cytokines such IL-1β, IL-6, and TNF-α were significantly higher in the sepsis group (Fig. 1C).

**Figure 2:**
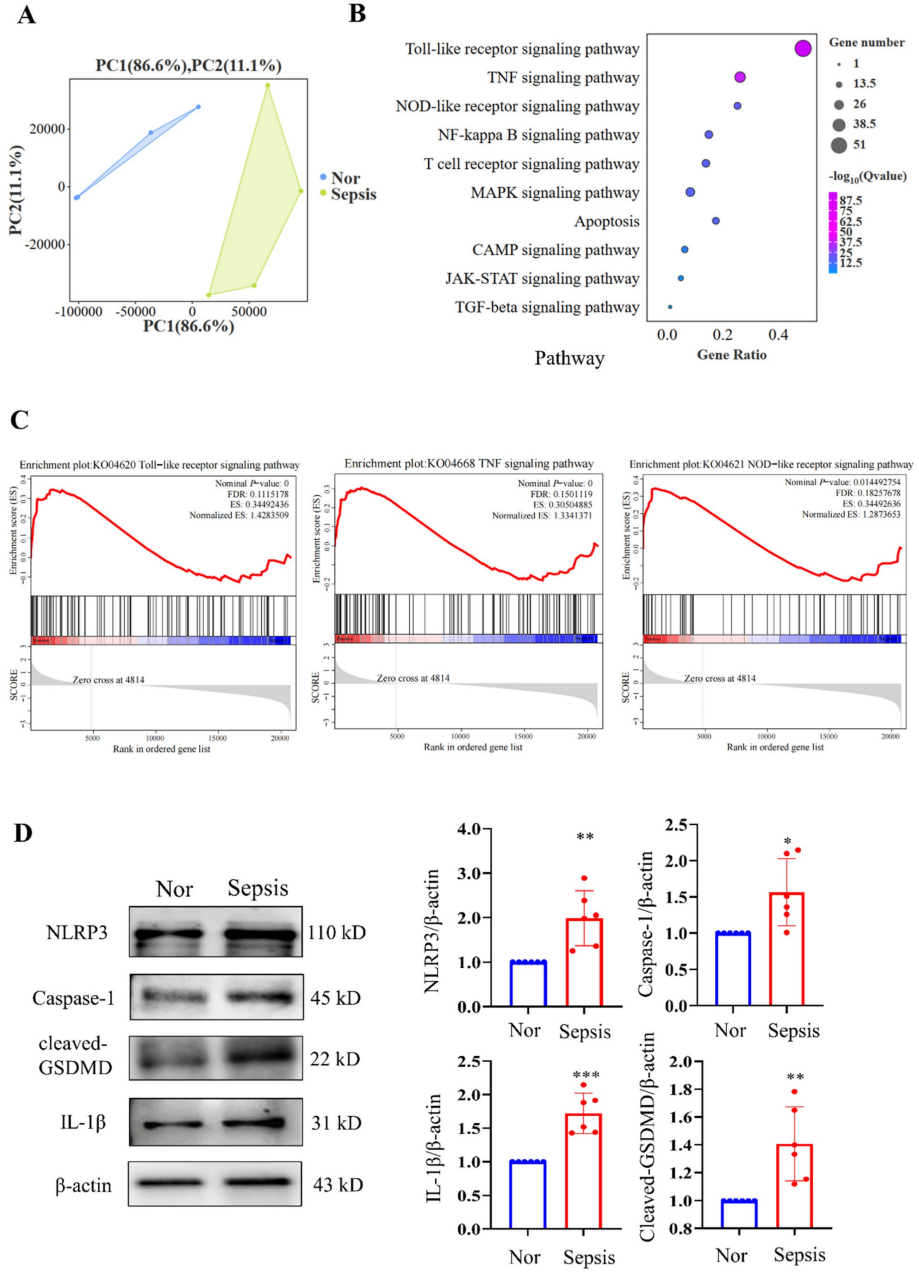
Sequencing analysis of PBMCs from healthy subjects and patients with sepsis. (A) PCA plot of PBMC samples from healthy volunteers and patients with sepsis (N=4). (B) KEGG analysis of the enriched pathways related to the cell cycle and death from transcriptome sequencing of PBMCs in healthy subjects and patients with sepsis (N=4). (C) GSEA of cell cycle and death-related pathways in the two groups according to KEGG analysis of DEGs from transcriptome sequencing of PBMCs in healthy subjects and patients with sepsis (N=4). (D) Western blotting of human PBMC samples to show the relative expression levels of NLRP3, caspase-1, cleaved-GSDMD, and IL-1β between the two groups. N=6. Data are shown as the mean ± SD. *P< 0.05, **P< 0.01 versus Nor. Nor: healthy volunteer.

We then performed RNA-seq analysis of PBMC samples from healthy volunteers and patients with sepsis and identified significant DEGs between them. Kyoto Encyclopedia of Genes and Genomes (KEGG) analysis revealed that (the PCA plot of all samples between the two groups is shown in Fig. 2A) the top three significantly enriched pathways were the Toll-like receptor signalling pathway, TNF signalling pathway, and NOD-like receptor signalling pathway, all of which were closely related to the progression of pyroptosis (Fig. 2B). GSEA of the gene sets for the Toll-like receptor, TNF, and NOD-like receptor signalling pathways revealed that these gene sets were highly enriched in the sepsis groups (Fig. 2C). Additionally, we discovered that NLRP3/caspase-1 signalling, which is a canonical pyroptosis pathway, was activated. The results demonstrated that PBMCs from the sepsis group had significantly higher relative protein levels of NLRP3, caspase-1, cleaved GSDMD, and IL-1β than those from the control groups (Fig. 2D). These results imply that sepsis results in pyroptosis in human PBMCs.

### Pyroptosis is activated in pulmonary macrophages in a CLP-induced septic mouse model and LPS-stimulated macrophages

**Figure 3:**
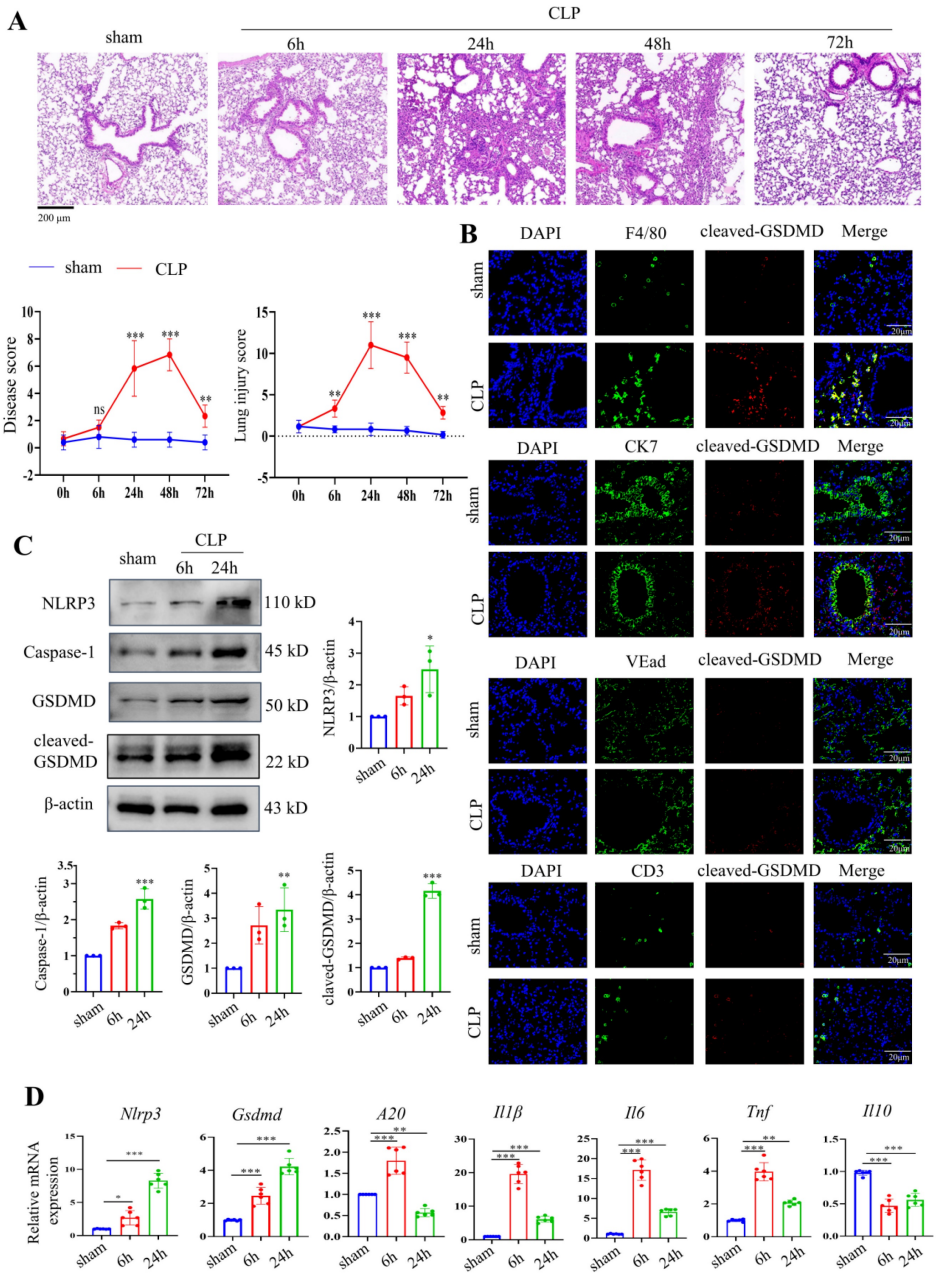
Activation of the NLRP3 inflammasome and pyroptosis in a mouse septic model. (A) Representative HE images of mouse lung tissues after CLP treatment over time. Scale bar means 200 μm. The disease score and lung injury score of lung tissues in each group were evaluated. N=6. (B) Double staining of F4/80 (red, macrophages), CK7 (red, epithelial cells), CD3 (red, T lymphocytes), VEad (red, endotheliocytes), and cleaved GSDMD (green) in the lung tissues of the sham and CLP-treated mice (24 h). DAPI is blue. N=6. Scale bar means 20 μm. (C) Relative protein expression levels of NLRP3, caspase-1, GSDMD, and cleaved GSDMD in the mouse lung tissues of the two groups as determined by Western blotting. N=3. (D) Relative mRNA expression levels of *Nlrp3*, *Gsdmd*, *A20*, *Il1β*, *Il6*, *Tnf*, and *Il10* in the mouse lung tissues of the two groups, as determined by qRT‒PCR. N=6. Data are shown as the mean ± SD. *P< 0.05, **P< 0.01, ***P< 0.001 versus the sham group.

A septic mouse model was induced by CLP operation. After CLP modelling over time, the lung tissues were stained using HE. CLP caused the alveolar structure to collapse and inflammatory infiltration in the lung tissues. Up to 24 hours after CLP, the damage gradually increased, was maintained for 48 hours, and then abruptly decreased (Fig. 3A). We also evaluated pathological changes in other organs, such as the heart, lung, liver, intestine, kidney, and spleen, by HE 24 hours after modelling, given that sepsis could cause damage to numerous organs. The results revealed that 24 hours after CLP, lung injury was the most severe when compared to that of other organs (Supplementary Fig. 1A). We then stained F4/80, CK7, CD3, and VEad in lung tissues following CLP with cleaved GSDMD to further investigate pyroptosis in immune cells in sepsis-induced lung damage (Fig. 3B). We then stained F4/80, CK7, CD3, and VEad together with cleaved GSDMD or TUNEL in lung tissues of CLP-treated mice to further investigate pyroptosis in immune cells in sepsis-induced lung damage. The enhanced staining for cleaved GSDMD and TUNEL in F4/80^+^ cells indicated that pyroptosis and apoptosis are more severe in macrophages. In addition, F4/80 and iNOS (an M1 marker) or CD163 (an M2 marker) were stained. In the CLP group, lung macrophage iNOS^+^ cells were clearly increased, indicating that CLP increased the M1 polarization of pulmonary macrophages (Supplementary Fig. 1B). Crucial molecules of the pyroptosis-related NLRP3/caspase-1 signalling pathway were detected in lung tissues 6 hours and 24 hours after CLP. NLRP3, caspase-1, GSDMD, and cleaved GSDMD protein levels were considerably higher in the lung 24 hours after CLP, according to Western blotting data (Fig. 3C). The mRNA levels of *Nlrp3* and *Gsdmd* were consistent with their protein alterations. Moreover, inflammatory indicators, including *A20*, *Il1β*, *Il6*, *Tnf*, and *Il10,* were identified in the mouse lung tissues by qRT‒PCR. Fig. 3D shows that CLP led to significantly increased levels of *A20*, *Il1β*, *Il6*, and *Tnf* 6 h after CLP, which were decreased 24 h after CLP. *Il10* levels were significantly decreased after CLP.

**Figure 4:**
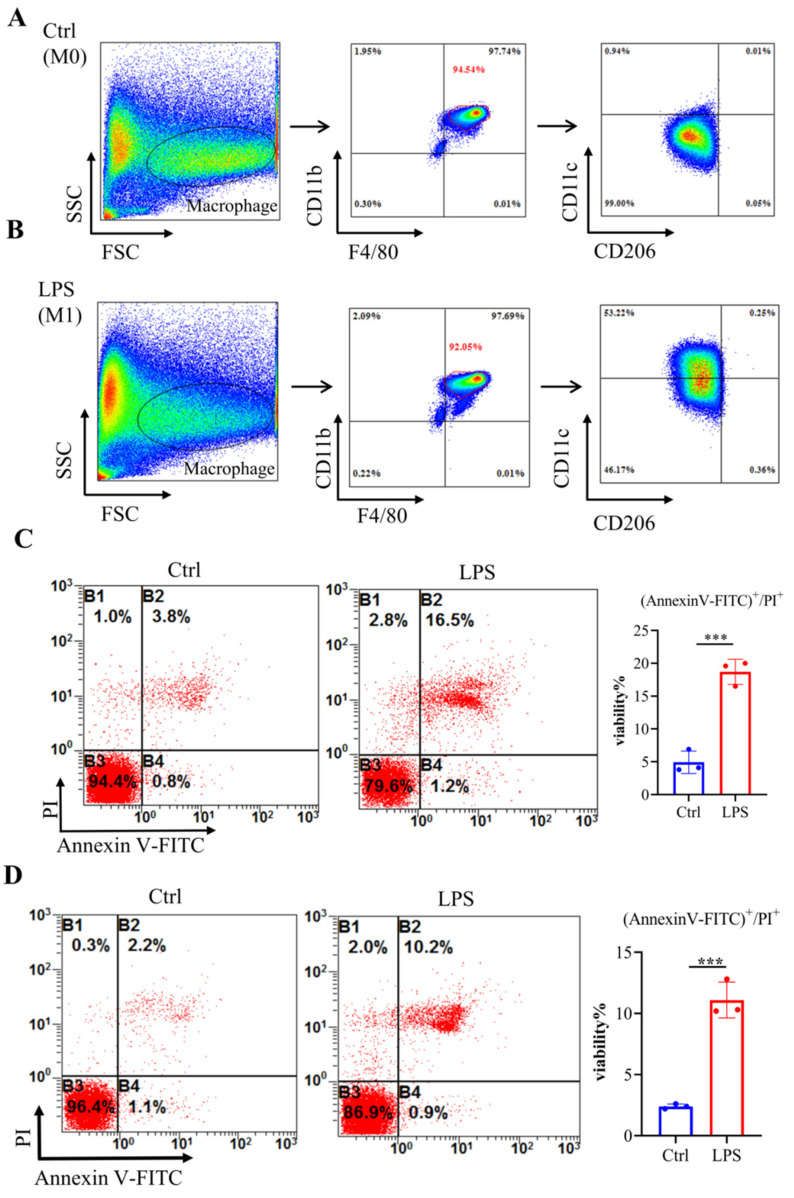
Bone marrow-derived macrophages (BMDMs) stimulated with LPS demonstrated an elevated level of cell death. (A-B) Flow cytometry analysis was used to analyse the polarization of BMDMs after LPS stimulation. N=6 in every group. BMDMs were first gated on FSC and SSC to remove debris and conjugates and then defined as CD11b^+^F4/80^+^ subpopulations (upper right), with the purity displayed as the percentage of the parent population gated on FSC/SSC. Tissue-infiltrated macrophages are defined as CD11b^+^F4/80^+^ cells; M1 macrophages are CD11b^+^F4/80^+^CD11c^+^CD206^-^ cells. (C) Annexin V/PI staining was used to identify and measure cell death in BMDMs following exposure to LPS. N=3. (D) Annexin V/PI staining was used to identify and measure cell death in RAW264.7 cells following exposure to LPS. N=3.

**Figure 5:**
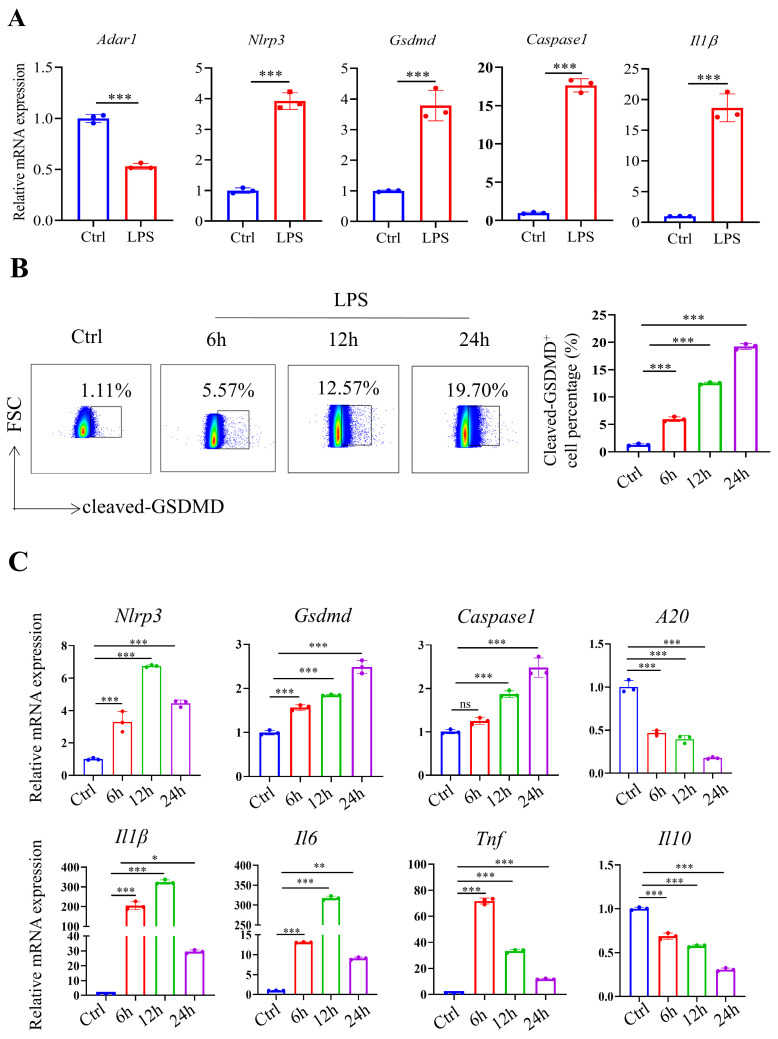
LPS-stimulated BMDMs and RAW264.7 cells exhibited increased pyroptosis. (A) The alterations in gene expression levels of *Adar1*, *Nlrp3*, *Gsdmd*, *caspase1*, and *Il1β* were assessed in BMDMs following 24 hours of LPS stimulation using qRT‒PCR. (B) Flow cytometry showing the percentage of cleaved GSDMD-positive cells in the control and LPS-treated (1 μg/ml) RAW264.7 cells. N=3. (C) qRT‒PCR analysis of *Nlrp3, Gsdmd, caspase1*, *A20*, *Il1β*, *Il6*,* Tnf*, and *Il10* levels in different cell groups*.* N=3. Data are shown as the mean ± SD. *P< 0.05, **P< 0.01, ***P< 0.001, ns=no statistical significance.

To mimic septic conditions in vitro, we stimulated BMDMs and RAW264.7 cells with 1 μg/ml LPS. Flow cytometry results showed that after LPS stimulation, BMDMs polarized towards M1 macrophages (Fig. 4A, 4B). Furthermore, Annexin-V/PI staining revealed a significant increase in cell death in both BMDMs and RAW264.7 cells after 24 hours (Fig. 4C, 4D). Furthermore, LPS stimulation led to a significant decrease in *Adar1* gene expression; the expression of *Nlrp3*, *Gsdmd*, *caspase1*, and *Il1β* was significantly upregulated (Fig. 5A). Cleaved GSDMD^+^ cells were significantly increased following LPS stimulation, as shown by flow cytometry (Fig. 5B). Further detection of CD11c and CD206 in LPS-treated cells showed that CD11c^+^ cells greatly increased, while CD206^+^ cells remained stable in RAW264.7 cells after LPS stimulation for 24 hours (Supplementary Fig. 1C). In addition, we performed qRT‒PCR to monitor changes in macrophage surface or metabolic markers to further validate the polarization status of RAW264.7 cells after 24 hours of LPS treatment. The results revealed that RAW264.7 cells predominantly polarized towards M1-type macrophages under LPS stimulation, despite not showing any statistically significant differences, and there was a slight increase in the expression of M2-type macrophage markers such *Arg1* and *Ym1* (Supplementary Fig. 1D). In addition, the relative mRNA levels of *Nlrp3*, *Gsdmd* and *caspase1* were markedly increased in LPS-stimulated cells. *Il1β*, *Il6*, and *Tnf* levels first increased and subsequently declined at 24 h following LPS stimulation, while *A20* and *Il10* were significantly decreased in LPS-treated cells (Fig. 5C). The findings demonstrate that in the lungs of septic mice produced by CLP and in LPS-stimulated RAW264.7 cells, macrophages underwent pyroptotic cell death.

### The expression of ADAR1 is downregulated in sepsis, while overexpressing ADAR1 facilitated immune reconstitution and alleviates pyroptosis in pulmonary macrophages of septic mice

**Figure 6:**
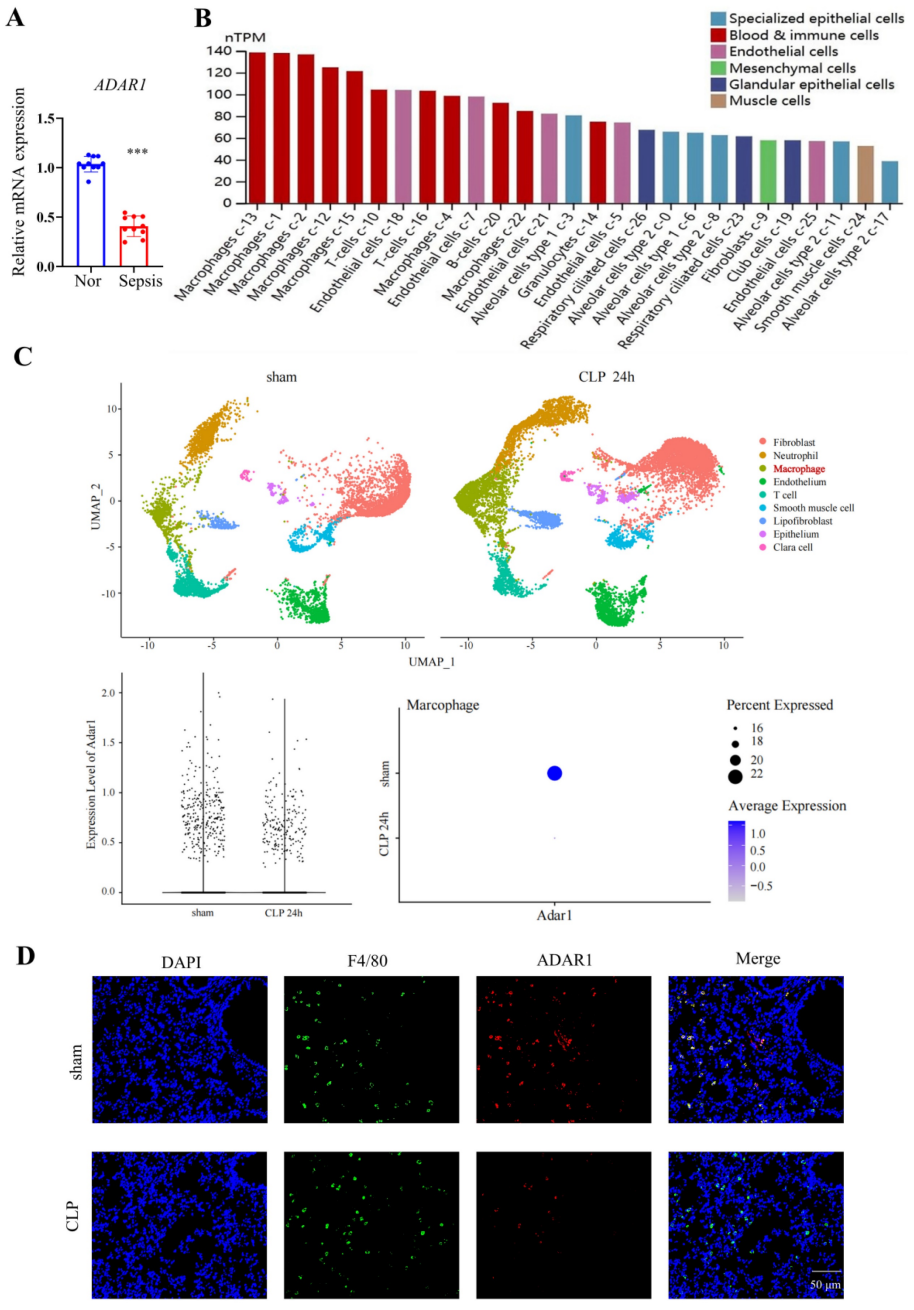
ADAR1 expression was decreased in septic CLP-treated mice. (A) ADAR1 mRNA expression levels in human PBMCs from healthy subjects (N=10) and patients with sepsis (N=10). ***P< 0.001 versus Nor. (B) The distribution and expression of ADAR1 in various cells in normal lung tissues. (C) ADAR1 expression in macrophages decreased 24 h after CLP. (D) Double staining of F4/80 (green) and ADAR1 (red) in the mouse lung tissues of the sham and CLP-treated mice. DAPI is blue. N=6. Scale bar represents 50 μm.

To investigate whether ADAR1 affects the pyroptosis of pulmonary macrophages in septic mice, the expression of ADAR1 in clinical sepsis (Fig. 6A) was evaluated. Results showed that ADAR1 was markedly reduced in PBMC of patients with sepsis. We analyzed the single-cell RNA sequencing data of human lung tissues to verify the distribution and expression of ADAR1 27. The results showed that while ADAR1 is extensively distributed in pulmonary cells in humans, macrophages express it at the highest level (Fig. 6B). In addition, we found that ADAR1 reduces in murine macrophages from a public database after 24 hours of CLP (Fig. 6C) and confirmed that lowered ADAR1 expression in lung macrophages in CLP mouse models (Fig. 6D).

**Figure 7:**
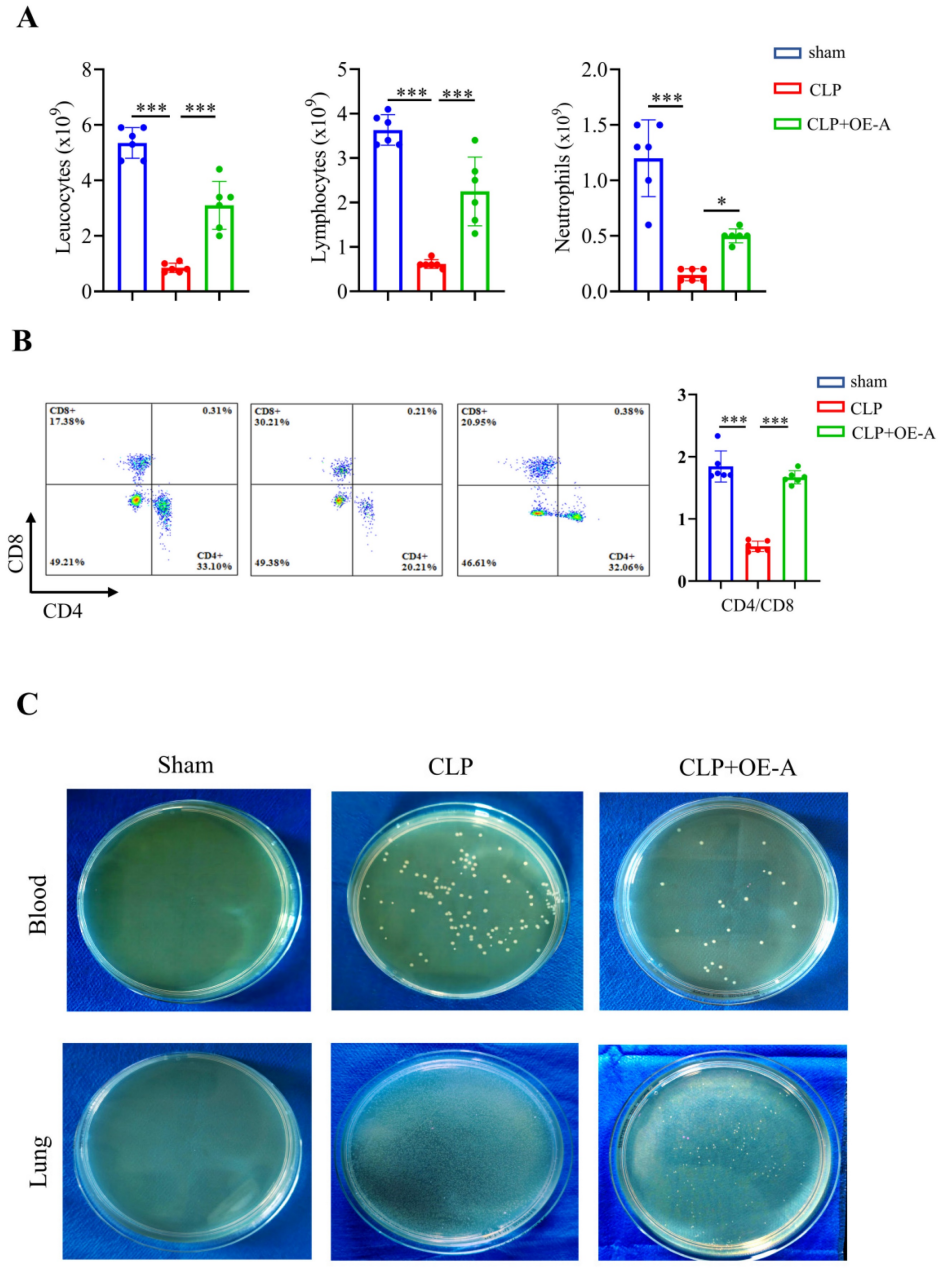
ADAR1 alleviates bacterial burden in septic mice, increases the CD4^+^/CD8^+^ ratio in peripheral blood, and promotes immune restoration. N = 6 in each group. (A) A comprehensive analysis was conducted on peripheral blood samples from mice in the sham, CLP, and CLP+OE-A groups to compare and evaluate the levels of leukocytes, lymphocytes, and neutrophils. (B) Flow cytometry was used to analyse the effect of ADAR1 on the CD4^+^/CD8^+^ ratio in mouse peripheral blood. (C) The levels of bacteria in blood and lung tissues after sham, CLP, and CLP+OE-A interventions using bacterial culture. The injection of the ADAR1 virus reduced the bacterial burden in the blood and lung compared to that in the CLP group. Data are shown as the mean ± SD. *P< 0.05, **P< 0.01, ***P< 0.001 versus CLP.

**Figure 8:**
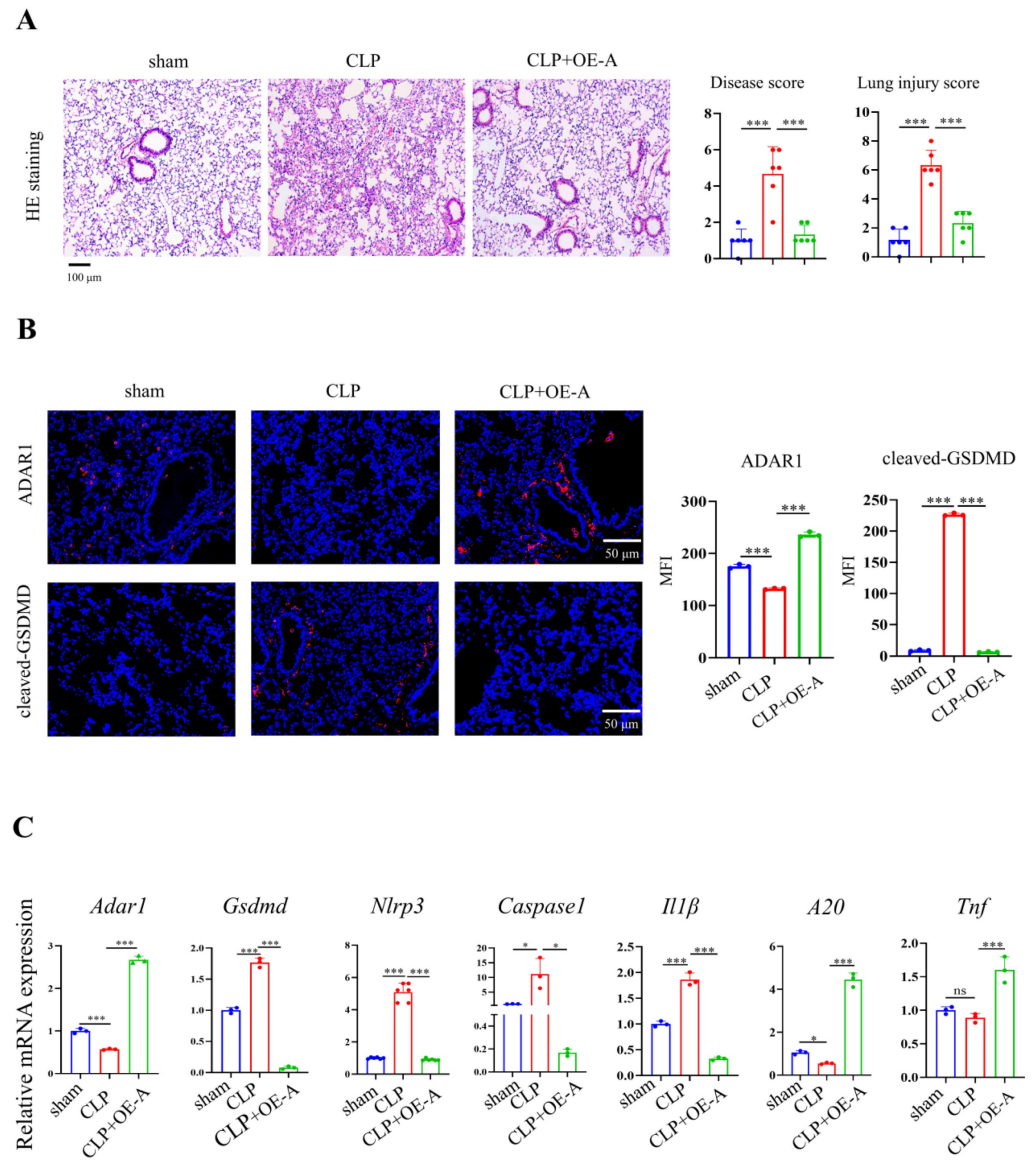
Overexpression of ADAR1 alleviated CLP-induced pyroptosis in mice. (A) HE staining of mouse lung tissues in the sham, CLP, and CLP with OE-A groups was performed. The disease score and lung injury score were evaluated. N=6. Scale bar represents 50 μm. (B) Immunofluorescence staining of ADAR1 (red) and cleaved GSDMD (red) in the mouse lung tissues of the sham, CLP, and CLP+OE-A groups. DAPI is blue. N=3. Scale bar represents 50 μm. (C) qRT‒PCR analysis of *Adar1*,* Nlrp3*,* Gsdmd*, *caspase1*, *A20*, *and Il1β* levels in different groups. N=3. Data are shown as the mean ± SD. *P< 0.05, **P< 0.01, ***P< 0.001, ns=no statistical significance.

Adenovirus expressing ADAR1 (OE-A) was injected into the tail vein of CLP-induced septic mice. The bacterial load test was conducted to evaluate the bacterial content in blood and lung tissues obtained from mice among the sham, CLP, and CLP+OE-A groups. The results showed that overexpressing ADAR1 could attenuate the bacterial load (Fig. 7C). Additionally, we conducted routine blood tests and flow cytometry to validate the effect of ADAR1 overexpression on sepsis-related immunoreaction. The remarkable updates in white blood cell count, lymphocyte count, and neutrophil count, along with the evident increase in the CD4^+^/CD8^+^ ratio observed in the CLP+OE-A group compared to the CLP group, provide compelling evidence for the crucial role of ADAR1 in promoting immune recovery, reducing bacterial burden, and exerting protective effects on septic mice (Fig. 7A, 7B). Moreover, HE staining of lung tissues was performed among the sham, CLP, and CLP+OE-A groups. ADAR1 overexpression alleviated CLP-induced lung injury in mice, according to both the disease scoring and lung injury scoring systems (Fig. 8A). We also detected the expression of ADAR1, cleaved GSDMD and TUNEL in the lung tissues among the three groups using immunofluorescence staining and found that the overexpression of ADAR1 resulted in significantly reduced cleaved GSDMD and TUNEL levels after CLP modelling (Fig. 8B, Supplementary Fig. 8B). The changes in ADAR1, cleaved GSDMD, and TUNEL were mainly in pulmonary macrophages based on double staining with F4/80 (Supplementary Fig. 2). The relative mRNA level of *Adar1* in the lungs was consistent with the above results. *Gsdmd*, *Nlrp3*,* Caspase1*, and *Il1β* showed significantly decreased mRNA expression levels in the CLP+OE-A group compared to the CLP group. In the lungs of CLP-treated mice, ADAR1 overexpression significantly increased the expression of A20 (Fig. 8C). These results suggested that ADAR1 is downregulated in sepsis, accompanied by an exacerbation of pyroptosis and apoptosis in macrophages; nevertheless, overexpression of ADAR1 reduces lung damage caused by CLP-induced activation of pyroptosis in lung macrophages (Fig. 8C).

### ADAR1 regulates pyroptosis and NLRP3 inflammasome activation in LPS-exposed RAW264.7 cells

**Figure 9:**
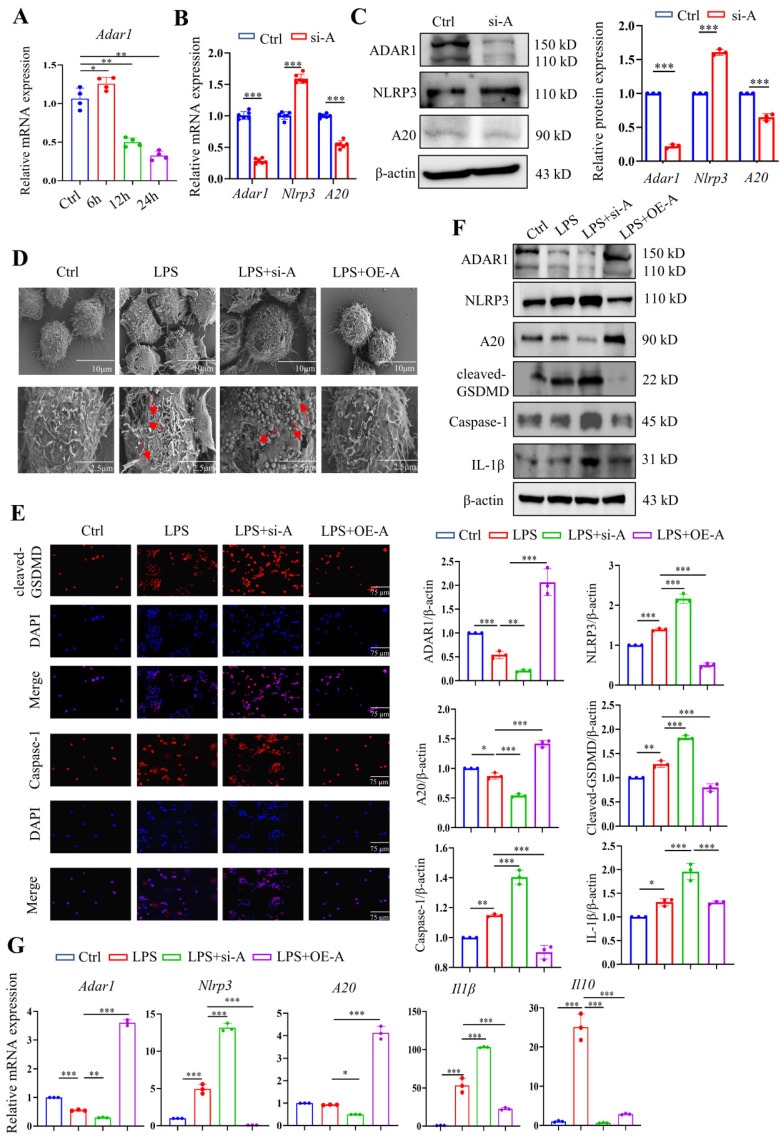
ADAR1 regulated pyroptosis in RAW264.7 cells after LPS stimulation through NLRP3 signalling. (A) The alteration of relative mRNA expression of *Adar1* in RAW264.7 cells with LPS stimulation over time. N=4. (B) RAW264.7 cells were transfected with ADAR1 siRNA (si-A), and the relative mRNA expression of *Adar1*, *Nlrp3*, and *A20* was measured by qRT‒PCR. N=6. ***P< 0.001 versus Ctrl. (C) Relative protein expression of ADAR1, NKRP3, and A20 was also assessed through Western blotting. N=3. (D) RAW264.7 cells were transfected with ADAR1 siRNA (si-A) and ADAR1 overexpression plasmid (OE-A) under LPS administration. SEM images showing the RAW264.7 cell morphology of the control, LPS, LPS+si-A, and LPS+OE-A groups. Scale bar indicates 10 μm and 2.5 μm. The red arrow indicates pore formation. N=6. (E) Immunofluorescence staining of cleaved GSDMD (red) and caspase-1 (red) in the four groups. DAPI is blue. N=6. Scale bar indicates 75 μm. (F) Western blotting images and quantification of NLRP3, A20, cleaved GSDMD, caspase-1, and IL-1β in the four groups. N=3. (G) Relative mRNA expression levels of *Nlrp3*, *A20*, and *Il1β* in the four groups determined by qRT‒PCR. N=3. Data are shown as the mean ± SD. *P< 0.05, **P< 0.01, ***P< 0.001.

The expression of *Adar1* in LPS-stimulated RAW264.7 cells was determined over time, and the results showed that it peaked at 6 h after LPS treatment and gradually declined until 24 h following LPS stimulation (Fig. 9A). Although A20 is well known as an anti-inflammatory molecule 38, it was also reported as a negative regulator of the NLRP3 inflammasome 39. RAW264.7 cells were then transfected with ADAR1-siRNA (si-A), and the relative mRNA and protein expression levels of ADAR1, NLRP3, and A20 were determined. ADAR1 and A20 were significantly decreased, and NLRP3 was markedly increased in si-A-transfected RAW264.7 cells (Fig. 9B, 9C). To verify the effects of ADAR1 on cell pyroptosis in septic macrophages, we transfected the cells with si-A or OE-A under LPS stimulation.

The highest levels of cleaved GSDMD were detected after 24 hours of stimulation, as previously reported; thus, we chose this time period for subsequent LPS treatment. We utilized scanning electron microscopy (SEM) to explore the morphological changes in each group. The results revealed that LPS clearly caused membrane pores and bulges, which were aggravated by ADAR1 knockdown but inhibited by ADAR1 overexpression (Fig. 9D). Immunofluorescence staining revealed that ADAR1 knockdown substantially increased cleaved GSDMD and caspase-1 levels in response to LPS, whereas ADAR1 overexpression significantly decreased them (Fig. 9E). According to the Western blotting results, ADAR1 deletion enhanced the LPS-induced activation of the NLRP3/caspase-1 signalling pathway, which was conversely alleviated by ADAR1 overexpression. Additionally, ADAR1 upregulated A20 expression in LPS-treated RAW264.7 cells (Fig. 9F). *Nlrp3*, *A20*, and *Il1β* relative mRNA levels were determined, and the results were consistent with their protein levels (Fig. 9G).

### ADAR1 regulates pyroptosis in macrophages through the miR-21/A20/NLRP3 signalling pathway

**Figure 10:**
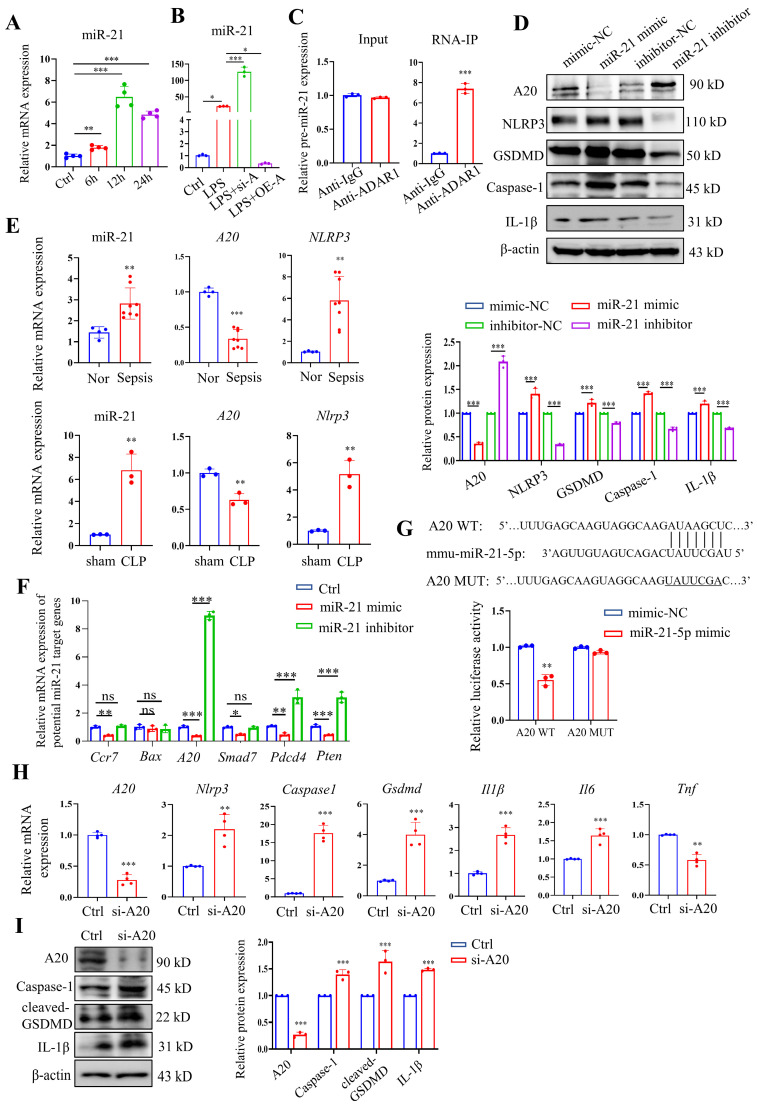
ADAR1 regulated NLRP3 inflammasome activity through the miR-21/A20 signalling pathway in sepsis. (A) The alteration of relative mRNA expression of miR-21 in RAW264.7 cells with LPS stimulation over time. N=4. (B) Relative expression of miR-21 in RAW264.7 cells in the control, LPS, LPS+si-A, and LPS+OE-A groups. N=3. (C) RIP assay showing the binding effects of ADAR1 with pre-miR-21 in RAW264.7 cells through isolation of RNA‒protein binding complexes via anti-ADAR1 and further qRT‒PCR measuring the pre-miR-21 levels. N=3. ***P< 0.001 versus anti-IgG. (D) RAW264.7 cells were transfected with miR-21 mimic or inhibitors, and the relative protein levels of A20, NLRP3, GSDMD, caspase-1, and IL-1β were assessed using Western blotting. N=3. (E) Above: relative mRNA levels of miR-21, *A20*, and *NLRP3* in PBMCs from healthy subjects (N=4) and patients with sepsis (N=8). **P< 0.01, ***P< 0.001 versus Nor. Below: relative mRNA levels of miR-21, *A20*, and *Nlrp3* in mouse lung tissues of the sham and CLP groups. N=3. **P< 0.01 versus sham. (F) Relative mRNA expression levels of several potential miR-21 target genes in RAW264.7 cells transfected with miR-21 mimic or inhibitors are shown. The genes related to pyroptosis and inflammation were screened from TargetScan. N=3. (G) Dual luciferase activity assay showing the direct binding effects of miR-21-5p with its target gene A20 in RAW264.7 cells. N=3. **P< 0.01. (H) Relative mRNA expression levels of *A20, Nlrp3*, *caspase1*, *Gsdmd*, *Il1β*, and* Il6* in Raw264.7 cells transfected with A20 siRNA (si-A20) determined by qRT‒PCR. N=4. (I) Western blotting of A20, caspase-1, cleaved GSDMD, and IL-1β in Raw264.7 cells transfected with si-A20. N=3. Data are shown as the mean ± SD. *P< 0.05, **P< 0.01, ***P< 0.001 versus Ctrl. ns=no statistical significance.

Our previous study revealed that ADAR1 regulated miR-21 biogenesis through its A-to-I RNA-editing effects on pre-miR-21 22. Here, miR-21 was clearly upregulated in RAW264.7 cells 24 h after LPS treatment (Fig. 10A). We also found that ADAR1 knockdown greatly elevated the relative expression of miR-21 in LPS-exposed RAW264.7 cells, whereas ADAR1 overexpression markedly decreased its levels (Fig. 10B). The direct binding effects of ADAR1 on pre-miR-21 were validated by an RNA-IP assay (Fig. 10C). We further transfected RAW264.7 cells with miR-21 mimic or inhibitor (the effects of the transfection were assessed using qRT‒PCR) and detected the activation of pyroptosis-related NLRP3/caspase-1 signalling through Western blotting (Fig. 10D) and qRT‒PCR (Supplementary Fig. 3). The results showed that the miR-21 inhibitor elevated A20 but decreased the levels of NLRP3, GSDMD, caspase-1, and IL-1β in RAW264.7 cells. In PBMCs from patients with sepsis, the mRNA levels of miR-21 and *NLRP3* were significantly increased, and the mRNA level of *A20* was decreased (Fig. 10E). These alterations were consistent with their expression in the lungs of CLP-treated mice (Fig. 10E). We further identified several potential miR-21 target genes that are related to the progression of cell pyroptosis using TargetScan and detected their mRNA levels in RAW264.7 cells transfected with miR-21 mimic or inhibitor. The results revealed that among these genes, A20 had the most significant change in the cells by miR-21 regulation (Fig. 10F). A dual-luciferase assay in RAW264.7 cells was used to confirm that miR-21-5p binds to the 3'UTR of A20 (Fig. 7G). In addition, the cells were transfected with A20-siRNA (si-A20), and downstream NLRP3/caspase-1 signalling was detected. Knockdown of A20 significantly increased the expression levels of* Nlrp3*, *caspase1*, *Gsdmd*, *Il1β*, and* Il6* (Fig. 10H). Relative protein levels of caspase-1, cleaved GSDMD, and IL-1β were also increased after A20 knockdown (Figure 10I). The findings collectively demonstrated that ADAR1 regulates macrophage pyroptosis by modulating the miR-21/A20/NLRP3 signalling pathway under septic conditions.

## Discussion

Although the in-hospital mortality of patients with sepsis has declined in recent years, rehospitalization, cognitive impairment, and physical disability after sepsis remain crucial issues that affect patients' quality of life 40. In the development of sepsis, a life-threatening battle takes place between the pathogen and the host immune system. The widespread death of immune cells triggered by sepsis, subsequent to organ dysfunction, is closely linked to poor patient outcomes 41. Previous studies have revealed that uncontrolled immune cell death is a major factor in triggering immune suppression in the body, and it is also one of the reasons for the production of different types of death patterns in PBMCs during sepsis 41, 42. In the initial stages of sepsis, peripheral immune cells experience phenomena such as apoptosis, necroptosis, and pyroptosis as they resist pathogen invasion. Of these, monocytes/macrophages and neutrophils, serving as the primary line of defence, exhibit the most severe cell death during the early phase of sepsis. Monocytes/macrophages are capable of recognizing and clearing pathogens in response to signals released by infected cells, while neutrophils promote macrophage pyroptosis through the formation of extracellular traps, influencing inflammatory responses 43-45. Moreover, our previous research has demonstrated that pyroptosis and lytic death are the major types of death of monocytes and neutrophils, whereas apoptosis, autophagy, and iron death have a relatively minor impact on the surrounding environment 46. Therefore, in-depth investigations of the complicated host response induced by infection in sepsis have important implications for the treatment and prevention of sepsis. In the present study, we found that pyroptosis is a substantial cell death pattern of PBMCs from patients with sepsis, and pyroptosis-related pathways are also significantly enriched in the cells, which is manifested in the activation of the NLRP3 inflammasome and an increase in proinflammatory cytokines. In the lung tissues of mice with sepsis caused by CLP, this characteristic of human septic PBMCs was also confirmed. The lungs showed considerable increases in lung macrophages and inflammatory infiltration, especially 24 hours after CLP modelling. Additionally, LPS-stimulated murine RAW264.7 macrophages showed elevated pyroptotic indicators over time. Furthermore, bone marrow-derived macrophages exhibited high levels of pyroptosis and cell death after 24 hours of exposure to LPS. Sepsis is characterized by an unbalanced immune response between excessive inflammation and immune suppression 47. Pyroptosis, an inflammatory cell death pathway, dominates the cell death pathways associated with sepsis. Studies have revealed that moderate pyroptosis contributes to host defences against intracellular pathogens, but when pyroptosis is out of control, it is detrimental to host cells 8, 16, 48.

Pyroptosis has both positive and negative consequences in sepsis, and the negative effects of pyroptosis far outweigh its positive effects 49. Moreover, pyroptosis-induced release of IL-1β promotes the recruitment and activation of neutrophils and macrophages 50. In mouse septic models, neutrophil extracellular traps increased macrophage pyroptosis in the peritoneal cavity through HMGB1/RAGE/dynamin signalling 45. Notably, a few studies, either in clinical patients or animal models, have confirmed lymphocyte pyroptosis in sepsis 49. Studies on macrophage pyroptosis in sepsis thus provide more evidence for exploration of the occurrence and underlying mechanism of sepsis.

Moreover, ADAR1 is downregulated in sepsis. Animal septic models have shown that ADAR1 is mostly expressed in pulmonary macrophages. A previous study also demonstrated that ADAR1 was preferentially expressed in pulmonary macrophages in inflamed tissues, indicating that ADAR1 plays a role in the pathogenesis of lung injury 51. Remarkably, in a CLP-induced septic milieu, the downregulation of ADAR1 expression results in a decrease in the immune cell population and an increase in bacterial burden. Simultaneously, ADAR1 expression was markedly downregulated, while pulmonary macrophages and their pyroptosis were significantly increased. According to Charles et al.'s hypothesis, phagocytes (including neutrophils and macrophages) make two distinct cell fate decisions that are influenced by GSDMD-dependent IL-1β production 52. One is cell pyroptosis, which mediates the rupture of cell membranes and the release of IL-1β, ultimately culminating in cell death. The other is cell hyperactivation, in which phagocytes are driven to secrete cytokines of the IL-1β family and participate in cellular immunomodulatory activities. Cell hyperactivation balances the pyroptotic death fate and, to a certain extent, maintains the quantity of productive macrophages while lowering the expense of the inflammatory response 49. This effect partially explains why the current study's observations of an increase in macrophage numbers were concurrent with an increase in macrophage pyroptosis due to sepsis. Furthermore, due to the varying degrees of pyroptosis, the impact on the organism also varies. To elucidate the association between high expression of GSDMD in macrophages and cell death in this experiment, we performed immunofluorescence double staining using cleaved GSDMD, F4/80, and TUNEL. Currently, TUNEL staining is not only employed for apoptosis but also widely utilized as a method to detect any form of programmed cell death. Therefore, in our study, we employed TUNEL staining as a marker for cell death 53-55. We found that cleaved GSDMD and TUNEL were significantly upregulated in macrophages during the development of a CLP model and when LPS was added, indicating a substantial enhancement in pyroptosis and cell death within septic environments. In addition, relative ADAR1 expression changed over time in murine macrophages exposed to LPS. The expression of ADAR1 in macrophages was also impacted by LPS concentration 51. Previous studies showed that 200 ng/ml LPS stimulation of RAW264.7 cells resulted in a considerable rise in ADAR1 up to 48 hours later 23. The maximum mRNA expression of ADAR1 was observed in these RAW264.7 cells after six hours of stimulation with 1 g/ml LPS, and it drastically decreased 24 hours later. This result was consistent with that of similar treatment-treated alveolar macrophages 56.

In the current study, overexpression of ADAR1 significantly attenuated CLP-induced lung injury and its related activation of the NLRP3 inflammasome. The effects of ADAR1 on macrophage pyroptosis were verified in LPS-exposed RAW264.7 cells, and further experiments revealed that the miR-21/A20/NLRP3 signalling pathway was involved in the regulation of macrophage pyroptosis by ADAR1, impacting the cell survival state in sepsis. A20 was discovered to be a negative regulator of the activation of the NLRP3 inflammasome in the development of rheumatoid arthritis 57. Moreover, A20 is negatively regulated by miR-21, which promotes the NF-B pathway and the NLRP3 inflammasome in LPS-induced pyroptosis and septic shock 14. Our previous study demonstrated that ADAR1 modulated miR-21 biogenesis through its editing effects, which were involved in the regulation of macrophage polarization 22. Based on this evidence, we investigated the regulatory roles of the ADAR1/miR-21/A20/NLRP3 signalling cascade in macrophage pyroptosis in septic lungs. Of course, other regulatory pathways may be involved in the formation and progression of sepsis due to the properties of the RNA-editing enzyme ADAR1 and numerous downstream targeting molecules of miRNAs, which calls for further research and investigation.

Several septic biomarkers relevant to physiological indices have been proposed in the past few years; nevertheless, there has not been much progress in determining which of them have clinical value 58. The findings of molecular regulators in sepsis provide new fresh perspectives on sepsis treatment and prevention. Excessive pyroptosis during sepsis has more negative effects than positive effects; hence, pyroptosis-suppressing treatments for sepsis have been investigated. These treatments primarily target the pyroptotic products IL-1β, inflammasomes, caspases, and GSDM molecules 49. In addition, dysfunction of ADAR1 leads to autoimmune illnesses and type I interferonopathies, among other immune system abnormalities 59. Although studies have indicated that ADAR1 is critical in sepsis and acute lung injury 23, 56, there is little research on how ADAR1 affects cell pyroptosis in the lungs following sepsis. In this study, we found that ADAR1 regulated sepsis-induced pyroptosis in pulmonary macrophages via the miR-21/A20/NLRP3 axis, which could help guide future therapeutic interventions that target this pyroptotic signalling pathway in sepsis-induced lung injury.

## Conclusion

In sepsis-induced lung injury, ADAR1 demonstrated protective functions. Clinical samples and CLP mouse models both showed downregulation of ADAR1 in sepsis, and overexpression of ADAR1 significantly alleviated lung injury from CLP and reduced the activation of the NLRP3 inflammasome in pulmonary macrophages. The miR-21/A20/NLRP3 signalling cascade, which may be thought of as an intervention target for the treatment of sepsis-related lung disorders, was responsible for these results. However, additional clinical studies are needed to confirm the regulatory functions of ADAR1 in sepsis.

## Supplementary Material

Supplementary figures and table.

## Abbreviations

ADAR1: Adenosine deaminase acting on double-stranded RNA 1
ALI: Acute lung injury
ARDS: Acute respiratory distress syndrome
CK7: Cytokeratin 7
CLP: Caecal ligation and puncture
DEGs: Differentially expressed genes
GSDMD: Gasdermin D
HE: Haematoxylin and eosin
IF: Immunofluorescence
IHC: Immunohistochemistry
IL: Interleukin
IOD: Integrated option density
KEGG: Kyoto Encyclopedia of Genes and Genomes
BMDM: Bone marrow-derived macrophages
LPS: Lipopolysaccharide
MFI: Mean fluorescence intensity
miRNA: MicroRNA
MUT: Mutant
NF-κB: Nuclear factor kappa B
NLRP3: Nod-like receptor (NLR) family pyrin domain containing 3
PBMC: Peripheral blood mononuclear cell
qRT‒PCR: Quantitative reverse transcription-PCR
RIP: RNP immunoprecipitation
SD: Standard deviation
SEM: Scanning electron microscopy
WT: Wild type
