# Supplementary material for: ADAR1 protects pulmonary macrophages from sepsis-induced pyroptosis and lung injury through miR-21/A20 signaling
Source: Int J Biol Sci. 2024 Jan 1;20(2):464–85. doi: 10.7150/ijbs.86424 (PMC10758098; doi:10.7150/ijbs.86424)
Supplement: Supplementary file 1 — Supplementary figures and table. [file ijbsv20p0464s1.pdf]

Supplementary Material

Supplementary Figures

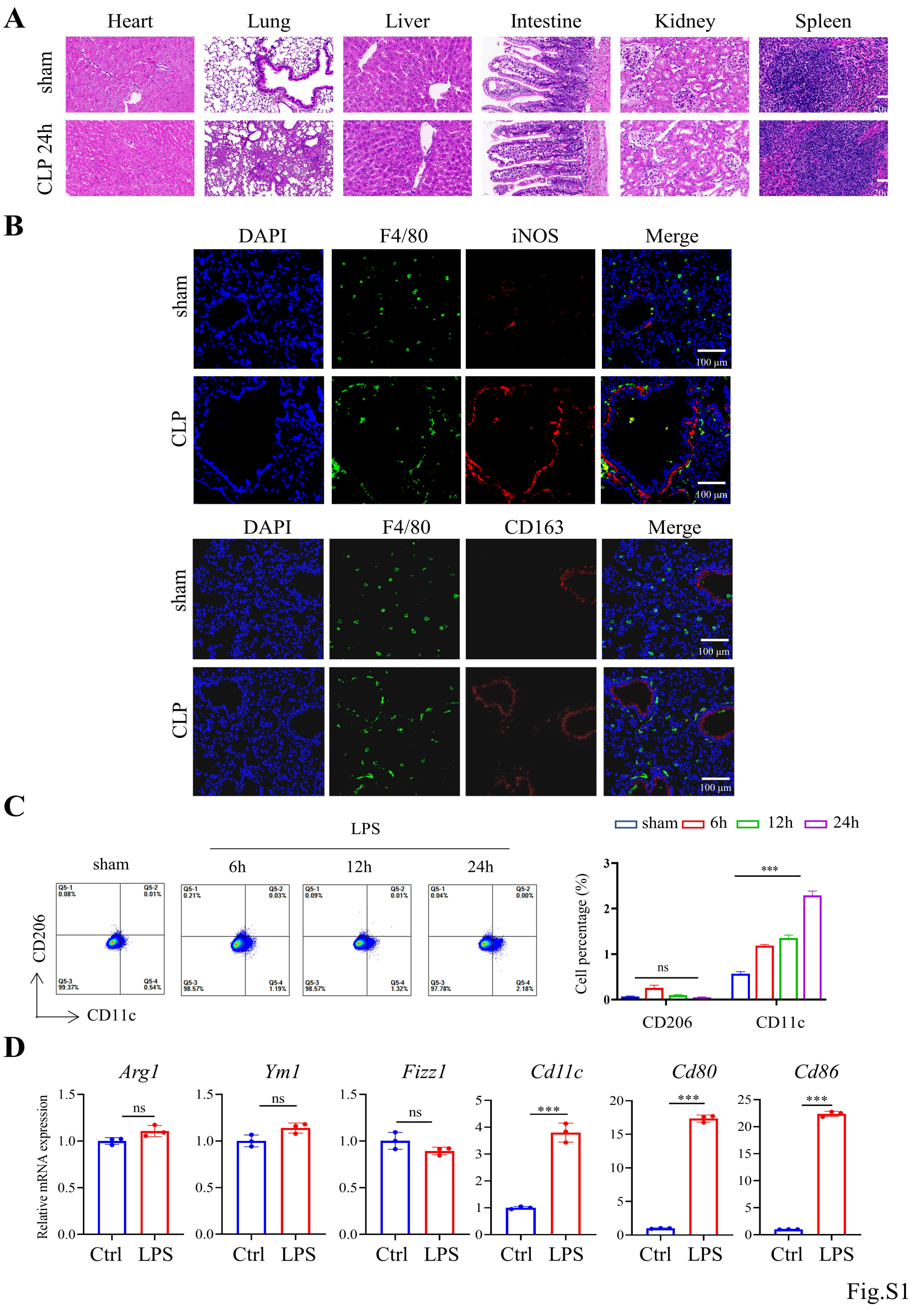

Supplementary Figure 1. CLP-induced organ pathological injuries in mice and macrophage polarization in lung tissues of CLP mice and RAW264.7 cells after LPS. (A), Representative HE images of mice heart, lung, liver, intestine, kidney, and spleen tissues after CLP treatment for 24 h. N=6. Scale bar means 20 μm. **(B),** Representative immunofluorescence images of double staining of F4/80 and iNOS (M1 marker) or CD163 (M2 marker) in murine lung tissues after CLP. N=6. Scale bar means 100 μm. **(C),** Flow cytometry of RAW264.7 cells with LPS stimulation over time showing cell percentage of CD11c (M1 marker) and CD206 (M2 marker) positive cells. N=3. **(D),** qRTPCR was used to assess the expression of M1 and M2 polarization in Raw 264.7 cells stimulated by LPS. N=3.Data were shown as mean ± SD. ****P*< 0.001, ns=no statistical significance (sham versus LPS-24h).

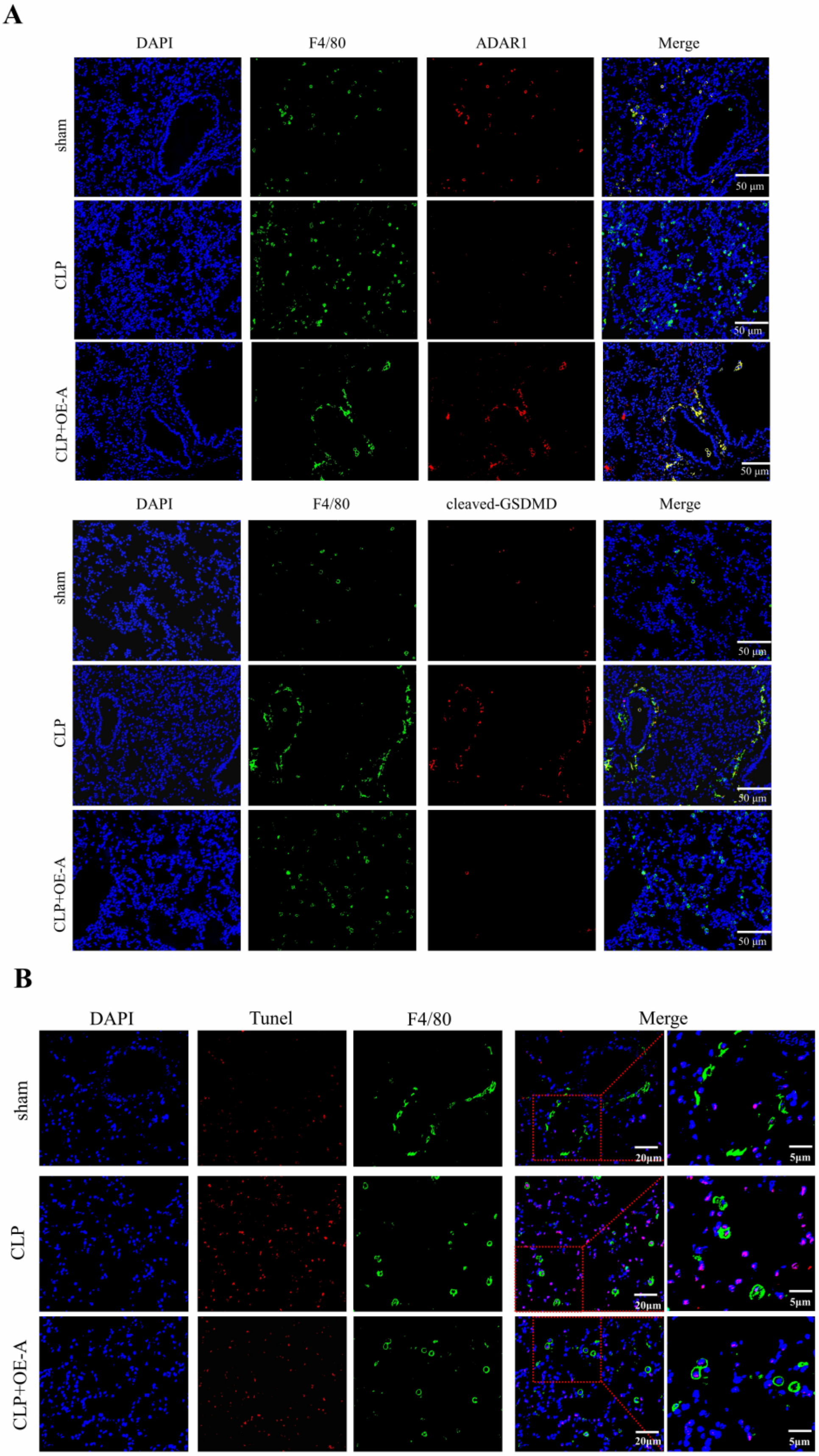

Supplementary Figure 2. Overexpression of ADAR1 alleviated CLP-induced pyroptosis and cell death in mice. (A), Double staining of F4/80 (green) and ADAR1 (red), as well as F4/80 (green) and cleaved-GSDMD (red) in murine lung tissues after CLP with or without ADAR1-overexpressing adenovirus infection. DAPI is blue. N=3. Scale bar means 50 μm.
(B), Immunofluorescence staining of tunel (red) and F4/80 (green) in the three groups. DAPI is blue. N=3. Scale bar means 20 μm.

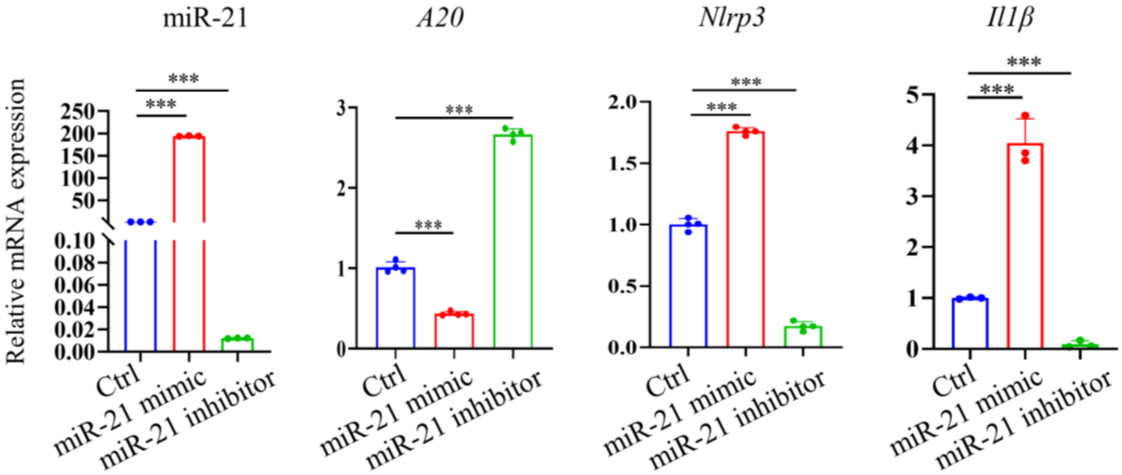

Supplementary Figure 3. ADAR1/miR-21/A20 signaling in RAW264.7 cells. RAW264.7 cells were transfected with miR-21 mimic or inhibitors, and relative mRNA levels of miR-21, A20, NLRP3, and IL-1β were detected via RT-qPCR. N=3. Data were shown as mean ± SD. *P< 0.05, **P< 0.01, ***P< 0.001.

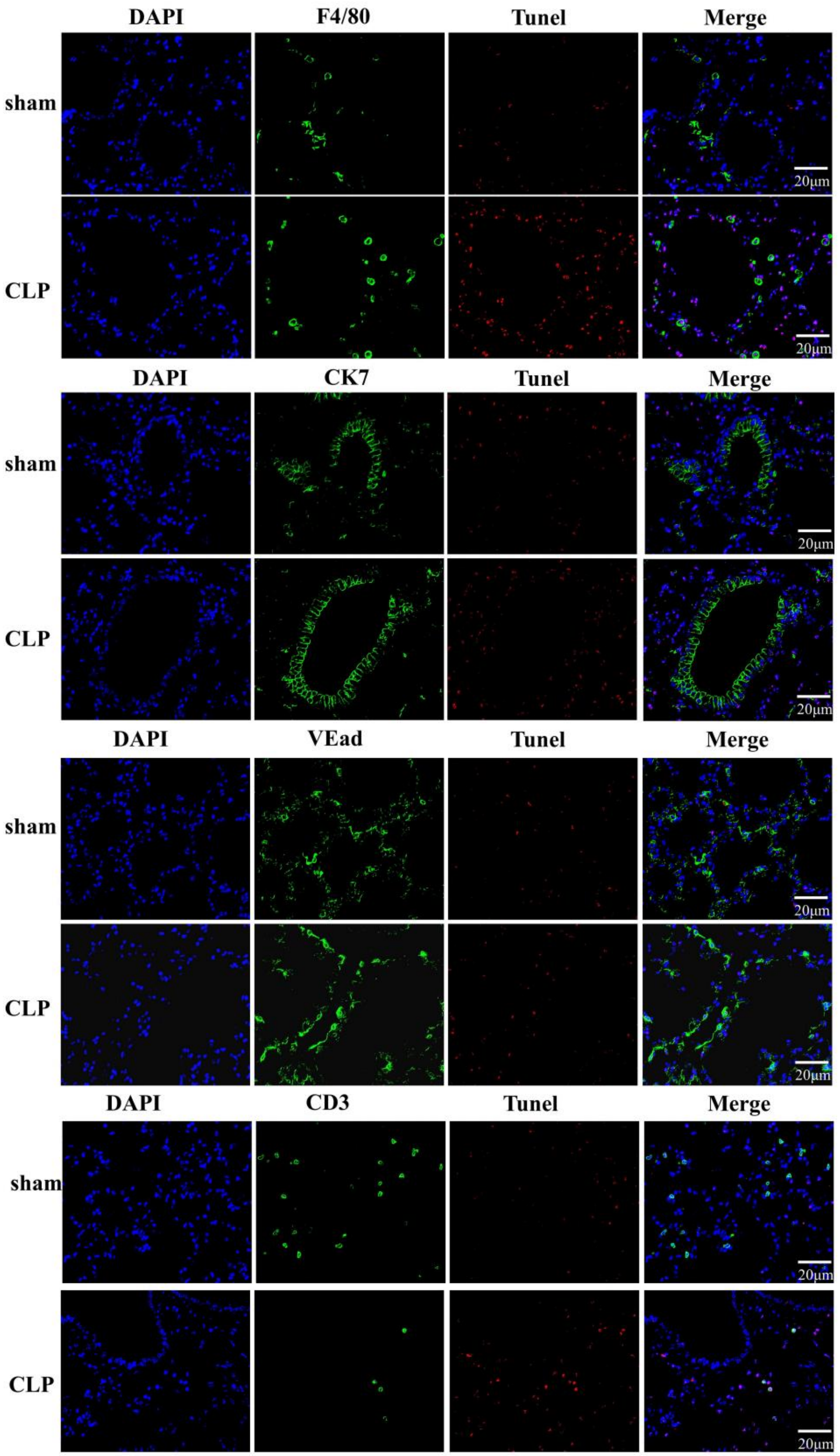

Supplementary Figure 4 Cell death of different cells in mice septic model. Double staining of F4/80 (green, macrophages), CK7 (green, epithelial cells), CD3 (green, T lymphocytes), VEad (green, endotheliocytes), and tunel (red) in the mice lung tissues of sham and CLP mice models (24 h). DAPI is blue. N=6. Scale bar means 20 μm.

Supplementary Table 1. Primer sequences used in RT-qPCR.

	Gene	Forward (5′-3′)	Reverse (5′-3′)
Human	*ADAR1*	CTGAGACCAAAAGAAACGCAGA	GCCATTGTAATGAACAGGTGGTT
	A20	CACACAAAGCACCTCAAGGC	GTTGGGATGCTGACACTCCA
	NLRP3	GATCTTCGCTGCGATCAACAG	CGTGCATTATCTGAACCCCAC
	*IL1*β	TGGCATTGATCTGGTTCATC	GTTTAGGAATCTTCCCACTT
	IL4	AACGGCTCGACAGGAACCT	CTCTGGTTGGCTTCCTTCACA
	IL6	GACAGCCACTCACCTCTTCA	TTCACCAGGCAAGTCTCCTC
	IL10	AGGGCACCCAGTCTGAGAACA	CGGCCTTGCTCTTGTTTTCAC
	GADPH	AGAAGGCTGGGGCTCATTTG	AGGGGCCATCCACAGTCTTC
Mice	*A*dar1	CCGTACCATGTCCTGTAGTGACA	GCCCTTGGCTGAAAAGGTAAC
	A20	GAACAGCGATCAGGCCAGG	GGACAGTTGGGTGTCTCACATT
	Nlrp3	TGGTGACTTTGTATATGCGTG	CTTAGGTCCACACAGAAAGT
	*I*l1β	CAGGATGAGGACATGAGCACC	CTCTGCAGACTCAAACTCCAC
	*I*l6	TGCTGGTGACAACCACGGC	GTACTCCAGAAGACCAGAGG
	*I*l10	GCCAGAGCCACATGCTCCTA	GATAAGGCTTGGCAACCCAAGTAA
	*T*nf	CGTCAGCCGATTTGCTATCT	CGGACTCCGCAAAGTCTAAG
	Caspase1	AGGCACGGGACCTATGTGAT	AGGGCAAAACTTGAGGGTCC
	Gsdmd	GATCAAGGAGGTAAGCGGCA	AACACTCCGGTTCTGGTTCT
	Smad7	AGGCTGTGTTGCTGTGAATCTTA	TGGGTATCTGGAGTAAGGAGGAG
	Pten	CATTGCCTGTGTGTGGTGATATC	TCCTCTGGTCCTGGTATGAAGAA
	Bax	ACCAGGGTGGCTGGGAAG	CCTTTCCCCTTCCCCCATTC
	Pdcd4	TCTGAAGCTGAACACTGCCTTAA	TGCACTTTCTCCAGTTGACTCTAA
	Ccr7	CAGGTGTGCTTCTGCCAAGAT	GGTAGGTATCCGTCATGGTCT
	Gapdh	GGTGAAGGTCGGTGTGAACG	CTCGCTCCTGGAAGATGGTG
